# Evaluation Protocols and Validation for Cameras in Indoor Healthcare Monitoring

**DOI:** 10.3390/s26175460

**Published:** 2026-08-28

**Authors:** Amirhossein Dadashzadeh, Jingjing Liu, Qianhui Men, Qiushuo Cheng, Kirsty Scott, Lisa Alcock, Ian Craddock, Majid Mirmehdi

**Affiliations:** 1 School of Engineering Mathematics and Technology, University of Bristol, Bristol BS8 1QU, UK; a.dadashzadeh@bristol.ac.uk (A.D.); qianhui.men@bristol.ac.uk (Q.M.); ian.craddock@bristol.ac.uk (I.C.); 2 School of Computer Science, University of Bristol, Bristol BS8 1QU, UK; qiushuo.cheng@bristol.ac.uk (Q.C.); m.mirmehdi@bristol.ac.uk (M.M.); 3 Translational and Clinical Research Institute, Faculty of Medical Sciences, Newcastle University, Newcastle upon Tyne NE1 7RU, UKlisa.alcock@newcastle.ac.uk (L.A.); 4 NIHR Newcastle Biomedical Research Centre, Newcastle University and The Newcastle upon Tyne Hospitals NHS Foundation Trust, Newcastle upon Tyne NE4 5PL, UK

**Keywords:** camera evaluation, RGBD cameras, depth accuracy, metrological validation, deployment factors, human pose estimation, indoor healthcare monitoring, MPJPE

## Abstract

Camera-based monitoring systems are increasingly adopted in healthcare settings for the continuous assessment of patient movement and activities. However, their technical performance under real-world indoor conditions remains insufficiently characterised, preventing appropriate selection when choosing cameras for clinical or home adoption and reproducibility. Existing validation studies typically assess either device metrological performance or algorithm accuracy in isolation, and often do not systematically account for practical deployment factors, such as lighting variability, occlusions, and camera positioning. To address this, we present two technical validation protocols that evaluate the same cameras at both the metrological and pose-estimation levels under systematically controlled deployment conditions rarely addressed together in prior work: the first evaluates the metrological performance of RGB and RGBD cameras, and the second assesses their use in supporting human pose estimation, validated using state-of-the-art pose estimators. The proposed protocols systematically assess five cameras (four RGBD and one RGB) under controlled variations in lighting, camera height, viewing angle, and occlusion level, within representative indoor scenarios. The experimental results show that metrological performance varies substantially across cameras, with depth bias at 5 m ranging from ∼10 mm to over 1400 mm depending on the device. For 2D pose estimation, all cameras achieve broadly comparable accuracy (mean mAP between ~78% and ~90%) across cameras and estimators, whereas 3D reconstruction error differs markedly across devices (MPJPE ranging from 104 mm to 365 mm), closely reflecting underlying depth sensing quality. Environmental factors have a camera- and estimator-dependent effect on 3D performance, while camera mounting height has minimal influence within the evaluated range. This work provides evidence-based guidance for the selection and deployment of cameras in healthcare monitoring applications, addressing an important gap in current technical validation practice.

## 1. Introduction

The ability to move safely and independently within indoor environments is a key determinant of health, autonomy, and quality of life, particularly for ageing populations and individuals with chronic conditions such as Parkinson’s disease. Changes in mobility and everyday movement patterns are closely linked to disease progression and functional decline, yet are often difficult to capture during short, supervised clinical assessments [1,2,3]. Continuous in-home monitoring therefore offers the potential to capture how people move during everyday activities in their own homes [2,4]. Such monitoring scenarios define the technical requirements considered in this study: unattended long-term operation in ordinary living spaces, with variable lighting, furniture occlusion, and everyday movements such as sit-to-stand and turning. We therefore incorporate these factors and movement tasks into our evaluation, assessing camera measurement quality before any application- or population-specific validation.

Existing approaches to movement assessment rely primarily on episodic clinical tests or wearable sensing technologies, such as inertial measurement units (IMUs), which have enabled quantitative evaluation of digital mobility outcomes in both laboratory and real-world settings [5]. While wearable sensors provide valuable local kinematic information, they are inherently limited in their ability to capture global spatial context, interactions with the environment, and complex indoor behaviours such as obstacle avoidance, turning around furniture, or sit-to-stand transitions performed in natural settings. Although IMUs can capture certain activities such as turning and sit-to-stand transitions, their ability to represent full-scene spatial context and interactions remains limited. In addition, long-term compliance and wearability can pose practical challenges for sustained monitoring in the home [6].

Camera-based monitoring systems provide a complementary sensing modality for indoor healthcare applications. By enabling markerless, passive observation, cameras can capture rich spatial and contextual information about human behaviour within indoor environments without requiring body-worn devices. Recent advances in computer vision, together with the widespread availability of low-cost RGB and RGBD cameras, have accelerated their adoption for applications, including gait analysis, symptom monitoring, and activity recognition and assessment, in home and clinical environments [7,8,9].

Despite this growing interest in at-home monitoring [8], the technical performance of such cameras under realistic indoor conditions remains insufficiently characterised, leading to poorly informed camera selection. Existing validation studies typically focus on one of two aspects: either to evaluate the metrological performance of camera devices, assessing properties such as depth accuracy, precision, stability, and field of view under controlled conditions [10,11,12] or to evaluate the accuracy of downstream vision algorithms—most commonly human pose estimation—by comparing derived kinematic or spatiotemporal measures against reference systems such as optical motion capture [13,14,15,16,17]. Although a few studies have attempted to combine both perspectives [18,19], they typically do not systematically account for practical deployment factors that strongly influence real-world performance, including lighting variability, camera placement, viewing angle, and occlusions. As a result, there is a lack of structured and reproducible technical validation protocols to support the reliable selection and deployment of consumer-grade cameras for indoor healthcare monitoring.

**Figure 1:**
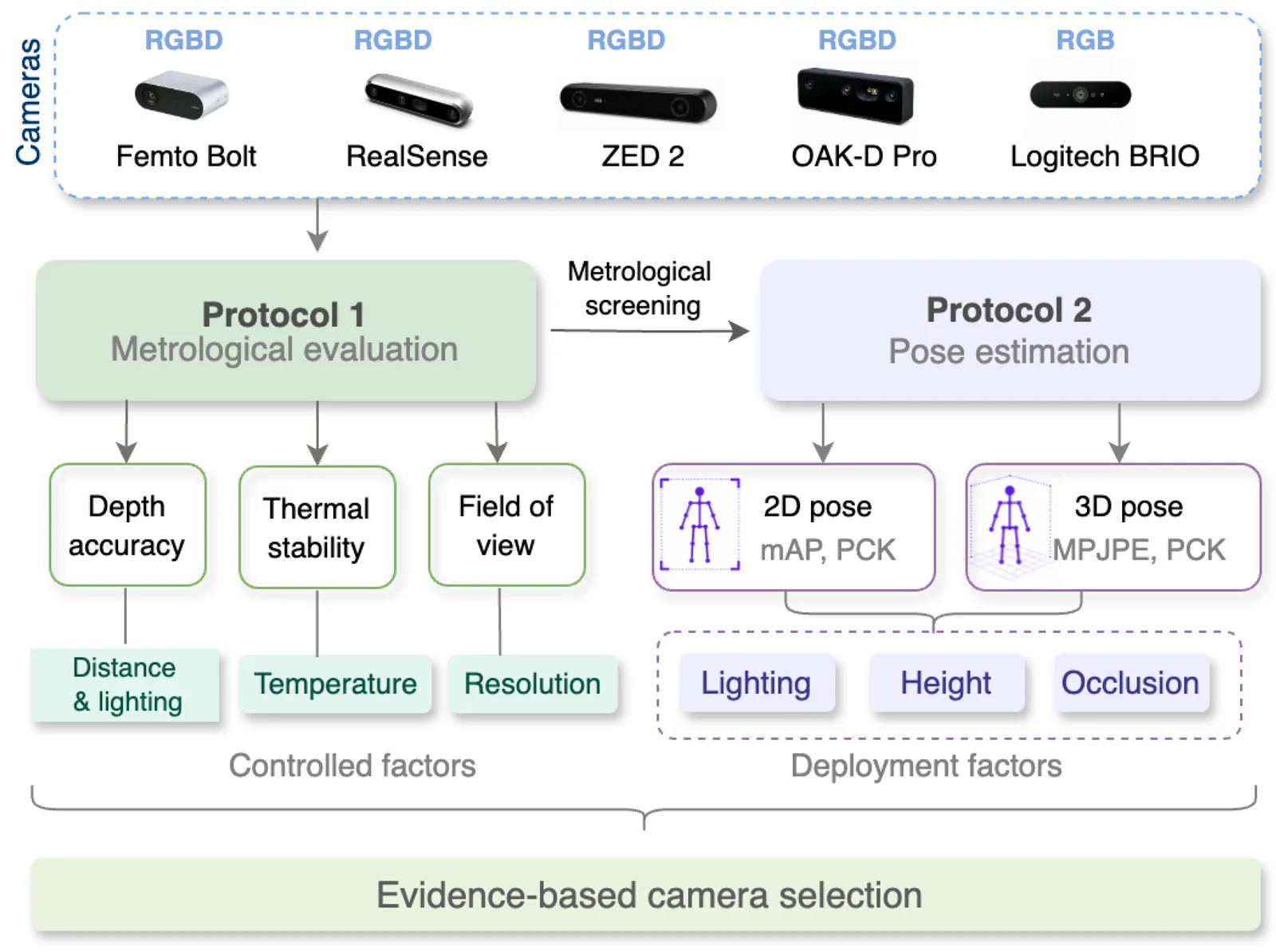
Overview of the proposed two-stage validation framework. Protocol 1 evaluates five cameras for depth accuracy, thermal stability, and field of view. This metrological screening determines which devices proceed to Protocol 2, which evaluates pose-estimation accuracy against motion capture under controlled indoor conditions. Together, the protocols link sensor-level capability with application-level performance to support evidence-based camera selection and deployment.

In this paper, we present comprehensive technical validation protocols for cameras that provide evidence-based guidance for their selection and deployment, particularly in indoor healthcare monitoring applications (see Figure 1). The framework follows a two-stage progression. Protocol 1 first characterises the sensor-level performance of five cameras (four RGBD and one RGB), including depth accuracy, field of view, thermal behaviour, and temporal stability, under controlled variations in distance, lighting, and ambient temperature. This metrological screening determines which devices proceed to Protocol 2. Protocol 2 then evaluates the selected cameras at the application level by assessing their reliability when integrated with human pose estimation under controlled indoor deployment conditions, including variations in lighting, camera placement (height and viewing angle), and occlusion. In this fashion, the two protocols link sensor-level measurement capability with downstream pose-estimation performance. For the pose-estimation stage, we considered several candidate estimators and focused on two architecturally distinct, real-time methods, RTMO [20] and YOLO26 [21]. Evaluating two structurally different algorithms allows us to examine whether the effect of camera characteristics on pose estimation is consistent across the types of algorithms that might be used in such applications, or whether it is estimator-specific.

The main contributions and technical novelty of this work are as follows:

- Two complementary validation protocols that evaluate the same cameras at both the metrological and pose-estimation levels under controlled deployment conditions, unlike prior work, which rarely combines both levels with systematic deployment-factor analysis.
- Validation of these protocols against a gold-standard motion capture system and architecturally distinct pose estimators.
- A systematically controlled experimental framework that quantifies the impact of key real-world deployment factors, including lighting conditions, camera placement (height and viewing angle), and occlusions.
- Comprehensive empirical analysis and evidence-based insights into the trade-offs between camera accuracy, robustness, and deployment practicality, supporting informed selection of sensing systems for indoor healthcare monitoring.

## 2. Related Work

Camera-based sensing has become an increasingly popular modality for indoor healthcare monitoring, driven by advances in computer vision and the availability of low-cost RGB and RGBD cameras [22,23,24,25,26,27]. Existing studies span a wide range of applications, as well as efforts to validate the reliability of camera-based measurements [10,12,14,28]. However, prior work is often fragmented, focusing either on application-level feasibility or on isolated aspects of camera performance. In this section, we review related literature from two complementary perspectives: camera-based indoor healthcare applications, and existing methodologies for validating camera performance.

### 2.1. Camera-Based Indoor Healthcare Applications

With the growing availability of consumer-grade cameras, many studies have explored using RGB and RGBD sensors as low-cost alternatives to marker-based motion capture systems [17,29]. Their use in healthcare research has reached not just clinical [22,24,25], but even home environments [4,23,26,27,30]. Among these applications, a variety of RGB cameras have been deployed, from  webcams [31,32], GoPro cameras [33], and cameras embedded in smartphones or mobile tablets [34,35] to systems such as the Microsoft Kinect [29], the Intel RealSense [36] and the ZED Stereo Camera [37].

In healthcare applications, camera sensors are typically used to capture people performing symptom-related motor tasks, including standardised assessments like the Timed Up and Go (TUG) test and gait trials. For instance, Dubois et al. [38] use a Kinect to automatically segment the TUG test into sit-to-stand, walking, turning, and stand-to-sit phases. Some studies track straight-line walking to estimate spatiotemporal and kinematic metrics such as step length, step width, gait speed, and joint range of motion for evaluating balance and mobility [39,40,41]. These metrics are usually derived by first recovering body skeletons from RGB or RGBD video using computer vision pose estimation (e.g., OpenPose [42] or Kinect skeletal tracking) and then computing clinically relevant gait and movement indicators.

While these studies demonstrate the potential of camera-based sensing for healthcare monitoring, several limitations remain. First, many existing works primarily focus on application-level feasibility, such as estimating gait parameters or segmenting functional tests, while the reliability of the underlying camera measurements is often assumed rather than systematically validated. Second, although some studies deploy cameras in real-world environments, the influence of environmental variability such as changes in lighting conditions and camera placement has rarely been systematically investigated. Third, different studies employ heterogeneous hardware platforms and pose estimation pipelines, making it difficult to directly compare measurement accuracy across systems. These limitations highlight the need for systematic methodologies to evaluate the performance of camera systems used for indoor healthcare monitoring.

### 2.2. Existing Camera Validation Methodologies

Existing camera validation studies can be broadly categorised into two research directions. The first focuses on the metrological performance of a device, i.e., the evaluation of its measurement properties such as accuracy, precision, repeatability, stability and geometric fidelity under controlled conditions [10,12,43,44,45]. This scope of research aims to determine how well a camera, as a sensing instrument, can capture reliable information independent of downstream processing. The second direction examines the validity of features extracted from camera-derived data, where raw RGB or RGBD measurements are combined with pose estimation or motion analysis algorithms, and the resulting kinematic or spatiotemporal metrics are compared against gold-standard references [17,28,46,47]. These studies evaluate the overall validity of the sensing and algorithm pipeline in practical application scenarios. Next, we consider existing work in each of these two areas.

For RGBD cameras, an essential component of metrological performance evaluation lies in validating the depth measurement quality. As summarised in Table 1, prior studies [10,11,12,43,45] commonly assess depth sensing through a set of fundamental metrological indicators, including bias, precision, accuracy, point-cloud quality as well as additional factors such as edge precision and 3D reconstruction accuracy. While the evaluated metrics differ across studies, the experimental conditions are much less diverse. Most studies assess camera performance primarily by varying the camera-to-object distance, whereas only a few additionally investigate illumination [10,11], typically under simplified lighting settings (e.g., dark or bright environments). For indoor healthcare monitoring, however, more representative lighting conditions, including normal, low, and very low illumination, as well as other deployment-related factors such as thermal stability and field of view, have received little attention.

For RGB cameras, prior works [48,49,50] examined their metrological properties, including image quality, camera speed, lens distortion and low light performance. However, RGB imaging is a long-established and mature technology with well-developed evaluation protocols, and is often treated as a reliable visual input in computer vision systems. Nevertheless, it is inherently limited to 2D appearance and lacks explicit depth, leading to scale ambiguity in recovering real-world geometry. In healthcare applications such as human pose estimation and gait analysis, accurate geometric measurements (e.g., joint positions, distances, and motion amplitudes) are essential. RGBD cameras address this limitation by providing depth information for recovering absolute 3D geometry. Therefore, depth sensing becomes critical for quantitative evaluation, motivating systematic validation of depth measurement performance.

This study focuses on categories of cameras that are commercially available and widely adopted, with the proviso that the cameras should be practical and cost-effective for indoor activity monitoring. Consumer-grade 3D LiDAR cameras are very limited in availability. Among the few representative devices, one of the main contenders, i.e., the Intel RealSense L515, has reached end-of-life [51]. In the ToF-based category, the Microsoft Kinect v2 has been discontinued, and we examine the Orbbec Femto Bolt as a now more commonly adopted ToF camera. Consequently, this work focuses on representative RGBD camera categories, including active stereo (Intel RealSense D456 and OAK-D Pro), passive stereo (ZED2), and ToF (Femto Bolt), together with a representative consumer-level RGB camera (Logitech Brio 4K).

Beyond metrological evaluation of cameras, a second line of research [18,19] focuses on the concurrent validation of camera-derived data and the algorithms applied to them. These studies typically rely on an additional reference measurement system to establish ground truth. Early work [52,53] adopted a single wearable IMU as the reference, which enabled only partial assessment of small range segment motions, such as limited upper-body orientation or trunk/pelvis angular parameters. As validation methodologies matured, researchers increasingly employed optical motion-capture systems such as Vicon [46], Qualisys [54], or OptiTrack [55], which can capture full-body 3D trajectories at high temporal and spatial resolution and thus serve as the gold standard for evaluating vision based measurements.

Using these reference systems, studies have validated various forms of camera-derived human motion representations. Some works [13,14] extract 2D keypoints directly from a single RGB camera using algorithms such as OpenPose [42] or DeepLabCut [56]. Others reconstruct 3D joint positions either through multi-camera triangulation of 2D poses [17] or by leveraging the depth information from RGBD sensors [15,16]. With the emergence of recent pose estimation methods such as [20,21], camera validation can now be performed using substantially more accurate and efficient pose estimation pipelines than those adopted in earlier studies. In addition, among these works, data are typically collected under controlled and repeatable movement scenarios, which may not fully capture the complexity and variability of human movements encountered in real-world healthcare environments. Most commonly, participants walk along a straight overground path [13,16,57] or on a treadmill [17] at self-selected or fixed speeds, enabling the extraction of spatiotemporal and kinematic gait parameters. Other studies [15] focus on static posture assessment, capturing images from multiple viewpoints—such as frontal, lateral, and posterior perspectives.

Based on these estimated 2D or 3D poses, researchers compute clinically relevant metrics, particularly those related to neurological and musculoskeletal conditions, including Parkinson’s disease [34,35], polyneuropathy [16], stroke [13], and low-back pain [53]. Commonly assessed parameters include spatiotemporal gait features (step length, gait speed, cadence, stance/swing times) [46,47], joint kinematics (hip/knee/ankle range of motion) [17,57] and balance-related measures [52]. To quantify agreement between camera-based estimates and the reference system, these studies adopt statistical metrics such as the intra-class correlation coefficient (ICC), Pearson or concordance correlation coefficients, root mean square error (RMSE), coefficient of multiple correlation (CMC), and Bland–Altman analysis.

Collectively, these studies demonstrate the feasibility of using low-cost RGB and RGBD cameras for clinical motion assessment, but several limitations remain. While some works have investigated the influence of camera placement or height [47,58], their evaluations are typically related to specific movements of interest, such as straight-line walking or falling. Their influence has not been considered in assessing common activities such as everyday activities encountered in real-world healthcare settings. Moreover, the performance of some RGBD cameras is sensitive to lighting conditions, yet the effect of illumination on human pose estimation has received limited attention. Most existing studies evaluate derived gait or joint kinematic parameters rather than validating the underlying pose data against ground-truth measurements, leaving a gap in understanding how accurately cameras capture raw human motion. Furthermore, while camera manufacturers provide technical specifications under idealised conditions, these measurements are often obtained under controlled laboratory conditions and may not reflect performance in real-world healthcare environments, where factors such as lighting, surface properties, and viewing angles can significantly affect sensing quality [12]. Therefore, independent validation under realistic conditions is necessary. Metrological studies provide fundamental sensor characteristics—they do not evaluate how sensing errors propagate to downstream healthcare tasks. Conversely, application-oriented studies validate end-task performance, but usually cannot distinguish whether observed errors originate from the camera hardware or the vision algorithms. Consequently, neither perspective alone provides sufficient evidence for selecting cameras for healthcare deployment. To address these limitations, we propose two technical validation protocols that assess multiple cameras and explicitly consider key factors that may affect camera-based motion measurements.

## 3. Methods

Indoor deployment of cameras (particularly at home for healthcare monitoring) is strongly affected by practical factors such as lighting variability, occlusions, and movement characteristics, which we treat as controlled experimental variables. Next, we propose evaluation protocols that (i) quantitatively characterise camera metrological performance and (ii) assess camera suitability for pose-estimation-based monitoring. These protocols provide a reproducible and application-oriented framework for benchmarking camera systems under clinically relevant indoor conditions.

### 3.1. Protocol 1: Evaluation of Metrological Performance

This protocol evaluates the metrological performance of camera systems across three complementary aspects: (i) depth measurement error, (ii) thermal behaviour and temporal stability, and (iii) field of view (FOV). The evaluated devices include four RGBD cameras (Femto Bolt, Intel RealSense D456, ZED2, and OAK-D Pro) and one RGB camera (Logitech BRIO 4K). The evaluated RGBD cameras employ different depth sensing technologies as shown in Table A1 of Appendix A.

**Figure 2:**
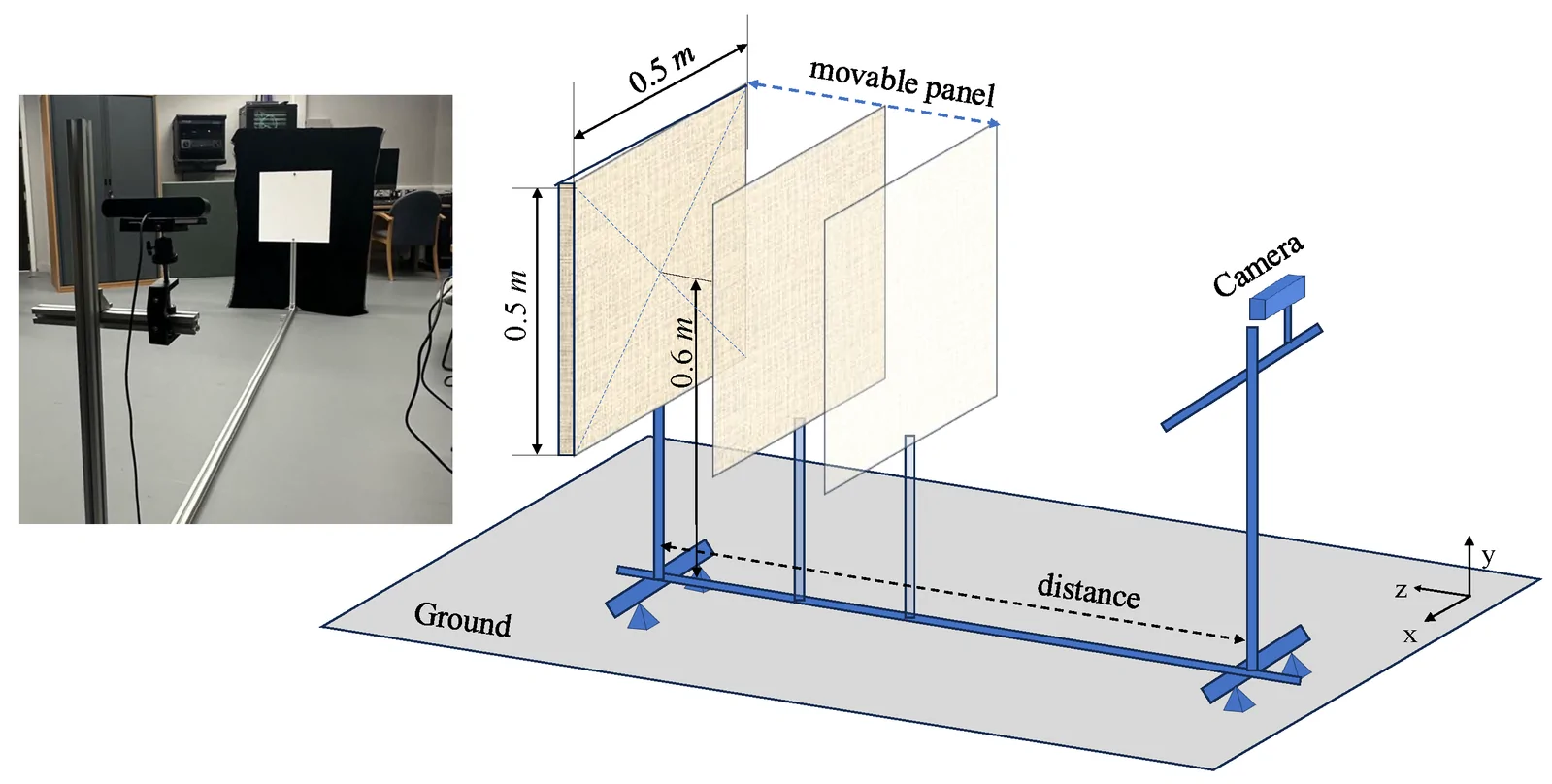
Experimental platform setup for measuring depth error.

**Depth measurement error—**Following established metrological evaluation methodologies for RGB-D cameras [10,11], this protocol assesses depth measurement error by measuring the distance between the camera and a planar target under controlled conditions. The target is positioned perpendicular to the camera’s optical axis *z* to ensure that measured depth values correspond directly to the camera–plane distance. The cameras and two laser range finders (RockSeed S2), used as ground-truth references, are mounted on construction profiles aligned in the xy plane. The laser range finders have a measurement accuracy of ±2 mm, according to the manufacturer’s specifications, providing an indication of the uncertainty associated with the reference distance measurements. A schematic of the experimental platform is shown in Figure 2. This configuration provides a controlled and repeatable setup for quantifying depth sensing accuracy across different camera devices. Depth measurements are evaluated under controlled variations of both lighting conditions and camera-to-object distance. Ambient illumination is monitored using a lux meter (URCERI MT-912) to ensure consistency across experiments. Under normal indoor lighting conditions (450–600 lux), depth is recorded at distances ranging from 1 to 5 m in 1 m increments. To assess the influence of reduced illumination, depths are measured under two scenarios: a low-light condition (50–100 lux) and a very low-light condition (3–10 lux). For both, measurements are again acquired at 1 to 5 m distances. For each combination of camera, lighting condition, and camera-to-target distance, 1000 frames are captured and used to calculate the depth measurement error.

Following [10], we use bias and precision to quantify depth measurement errors. The bias describes the systematic error of depth estimation, defined as

(1) $$\left|d_{gt}-\mu \right|,$$

with mean depth measurement μ as

(2) $$\mu =\sum_{i=1}^{n}\sum_{j=1}^{m_{i}}\frac{1}{n\cdot m_{i}}d_{i,j}.$$

$d_{gt}$ describes the ground truth distance, where *n* is the number of recorded frames, $m_{i}$ is the number of valid pixels on the current frame in the region of interest and $d_{i,j}$ denotes the corresponding depth measurement. Precision describes the random error of distance measurements and is defined as the standard deviation of distance measurements:

(3) $$precision=\sqrt{\sum_{i=1}^{n}\sum_{j=1}^{m_{i}}\frac{1}{n\cdot m_{i}}{\left|d_{i,j}-\mu \right|}^{2}}.$$

Depth bias is mainly caused by systematic factors such as sensor calibration offsets and systematic ranging errors, whereas depth precision is mainly affected by random noise, including sensor noise and environmental conditions.

#### 3.1.1. Thermal Behaviour and Temporal Stability

Thermal behaviour and temporal stability are critical for camera systems intended for continuous and unattended indoor monitoring, with measurement reliability affected over time due to temperature-induced sensor drift [59]. To characterise these, we evaluate camera stability by continuously operating each device for two hours while monitoring depth measurements and device temperature, with the target positioned at a fixed distance of 2 m.

Depth drift is quantified by measuring the variation in the mean depth value over time. At each recorded time step *t* (sampled at 30 fps), the mean depth $\bar{d}\left(t\right)$ is computed over a central region of interest consisting of 500 pixels, selected to minimise edge effects and represent the most stable portion of the depth image. Let $\bar{d}\left(0\right)$ denote the mean depth at the first recorded time step. Two complementary metrics are used to characterise depth stability over a total of *N* recorded time steps. We apply RMSE to calculate the overall deviation of the depth signal from the initial measurement,

(4) $$\mathrm{RMSE}=\sqrt{\frac{1}{N}\sum_{t=1}^{N}{\left(\bar{d}\left(t\right)-\bar{d}\left(0\right)\right)}^{2}},$$

and the average absolute drift (AAD) to quantify the mean magnitude of frame-to-frame depth variation:

(5) $$\mathrm{AAD}=\frac{1}{N-1}\sum_{t=1}^{N-1}\left|\bar{d}\left(t+1\right)-\bar{d}\left(t\right)\right|.$$

RMSE captures the cumulative deviation from the initial reference, reflecting both systematic drift and random noise, while AAD characterises the short-term frame-to-frame jitter of the depth measurement. Both metrics are recorded in millimetres. As electronic imaging sensors and signal-conditioning circuits are sensitive to temperature changes, these metrics provide indicators of thermally induced measurement instability.

Device temperature is monitored concurrently throughout each operating session using an external thermal camera (Sealey VS913). The maximum observed surface temperature of each device is noted as the representative device temperature at each time point. Three experimental factors are considered: scene dynamics, camera operation, and ambient room temperature. These are examined through two separate experimental configurations as described below.

*Scene dynamics and camera operation:* Cameras are deployed in the natural indoor environment of a home setting, where illumination and scene content may vary due to normal human activity. Cameras are operated continuously for two hours, during which a thermal camera records device surface temperature profiles.

*Ambient temperature conditions:* To isolate the effect of environmental temperature, cameras are mounted on the experimental platform at a fixed distance of 2 m from the planar target, with controlled lighting and no people in the field of view. An indoor air-conditioning system is used to regulate room temperature across three ranges: 18–20 °C (low), 23–25 °C (normal), and 28–30 °C (high). For each condition, cameras operate at their maximum supported frame rate and resolution, while both device temperature and depth drift are recorded over time.

#### 3.1.2. Field of View Angles

The FOV of a camera defines the angular extent of the observable scene and directly determines the spatial coverage achievable by the sensing system. A larger FOV enables broader scene coverage, which is particularly important for indoor monitoring scenarios where subject movement may span across large widths in the scene.

**Figure 3:**
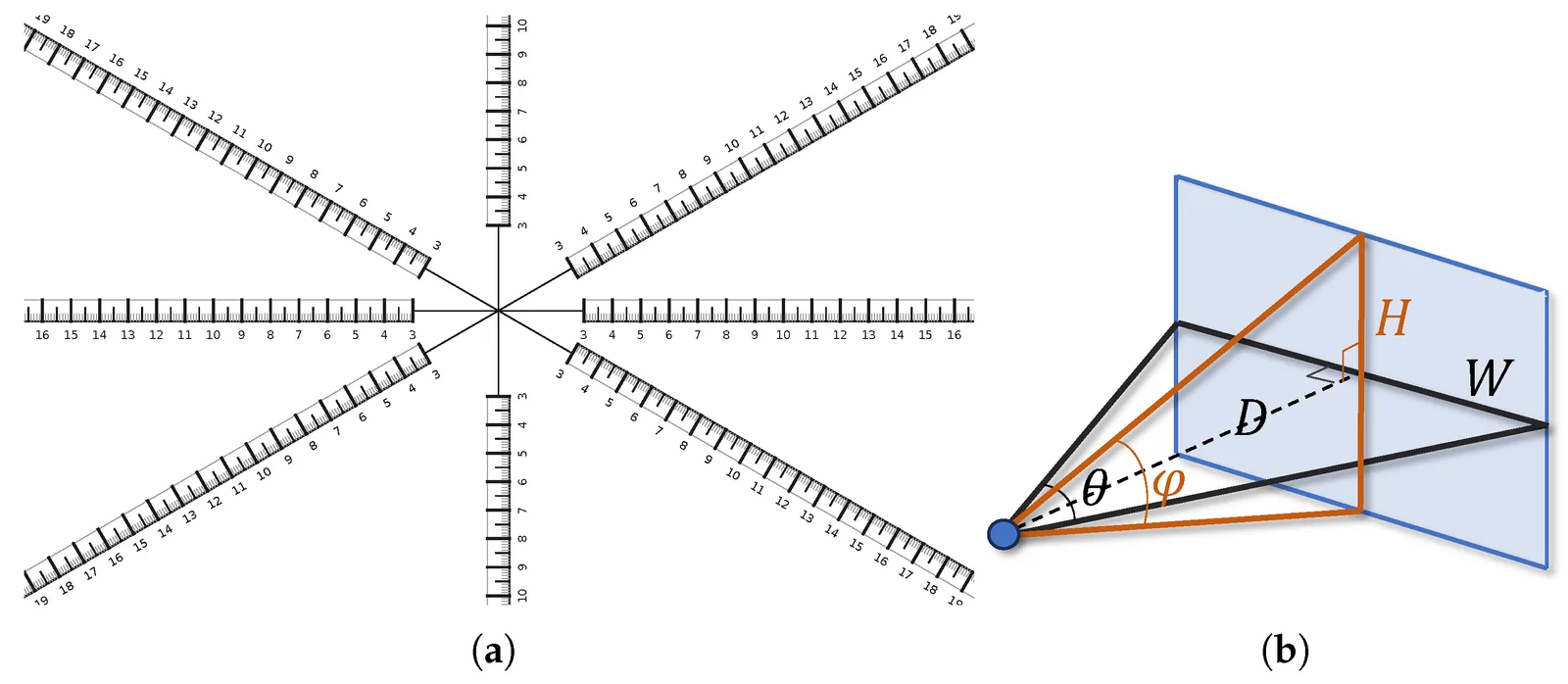
FOV angle measurements. (**a**) Acrylic sheet for FOV. (**b**) FOV angle calculations.

As illustrated in Figure 3a, an acrylic sheet with annotated dimensions is mounted on the planar surface of the experimental platform. The camera is carefully positioned to ensure that the image boundaries are aligned with the edges of the acrylic sheet and that the viewing direction is approximately perpendicular to the sheet. Images are acquired at a fixed shooting distance (e.g., 50 cm), which is measured using a laser distance metre. The horizontal and vertical extents of the captured area are then determined based on the ruler markings on the sheet. As shown in Figure 3b, the horizontal and vertical field-of-view angles are computed from the measured dimensions using

(6) $$\theta =2\tan^{-1}\;\left(\frac{W}{2D}\right),\;\varphi =2\tan^{-1}\;\left(\frac{H}{2D}\right),$$

where *W* and *H* denote the measured width and height of the visible area on the sheet, respectively, and *D* is the camera-to-plane distance. *D* was measured using the laser range finder with an accuracy of ±2 mm, while *W* and *H* were read from the scale on the acrylic sheet, with a minimum graduation of 1 mm. These specifications provide an indication of the measurement precision associated with the FOV calculation. The FOV measurement was performed once for each camera–resolution configuration, with measurement repeatability further discussed as a limitation in Section 5.

### 3.2. Protocol 2: Validation of Human Pose Estimation Performance

Participants are asked to perform various motion tasks while their movements are simultaneously recorded using cameras and a customised Vicon optical motion capture system comprising 8 Vantage v5 cameras and 10 Vero v2.2 cameras. This will facilitate quantitative comparison of pose estimation results against the mocap system, which serves as the reference measurement standard. The marker-based Vicon system is adopted as ground truth because it is metrologically independent of the markerless cameras under test, both in its sensing principle and in offering sub-millimetre accuracy, so that discrepancies can be attributed to the camera pipeline rather than a shared error source. This follows established practice in vision-based pose estimation, where optical motion capture provides the reference measurement in widely used benchmarks such as Human3.6M [60]. The Vicon system is calibrated before each recording session using the manufacturer’s standard calibration procedure. Calibration quality is verified by ensuring that the image reprojection error remains below 0.3 pixels for all cameras.

The following subsections describe the instrumentation, experimental setup, data collection, signal processing and synchronisation, spatial alignment of skeleton data, as well as the evaluation metrics used in this study.

#### 3.2.1. Instrumentation

Four cameras are evaluated for indoor human pose estimation, including three RGBD cameras, namely Femto Bolt, Intel RealSense D456, and ZED2, as well as one RGB camera, Logitech BRIO 4K. Following the depth evaluation experiment presented later, the RGBD camera OAK-D Pro was not included in other experiments, as it was found to exhibit substantially larger depth measurement errors compared with the other RGBD cameras. In addition, OAK-D Pro has the narrowest field of view among the evaluated devices, which prevents it from consistently capturing the full body in Protocol 2. The analysis of OAK-D Pro’s performance is shown in Section 4.1.

#### 3.2.2. Experimental Setup and Data Collection

Experiments were conducted with a single healthy male participant (age: 32 years; height: 176 cm; body mass: 84 kg). A single participant was used to ensure consistent body geometry and motion patterns across repeated trials, allowing the evaluation to focus on the measurement characteristics of the camera systems rather than inter-subject variability.

**Figure 4:**
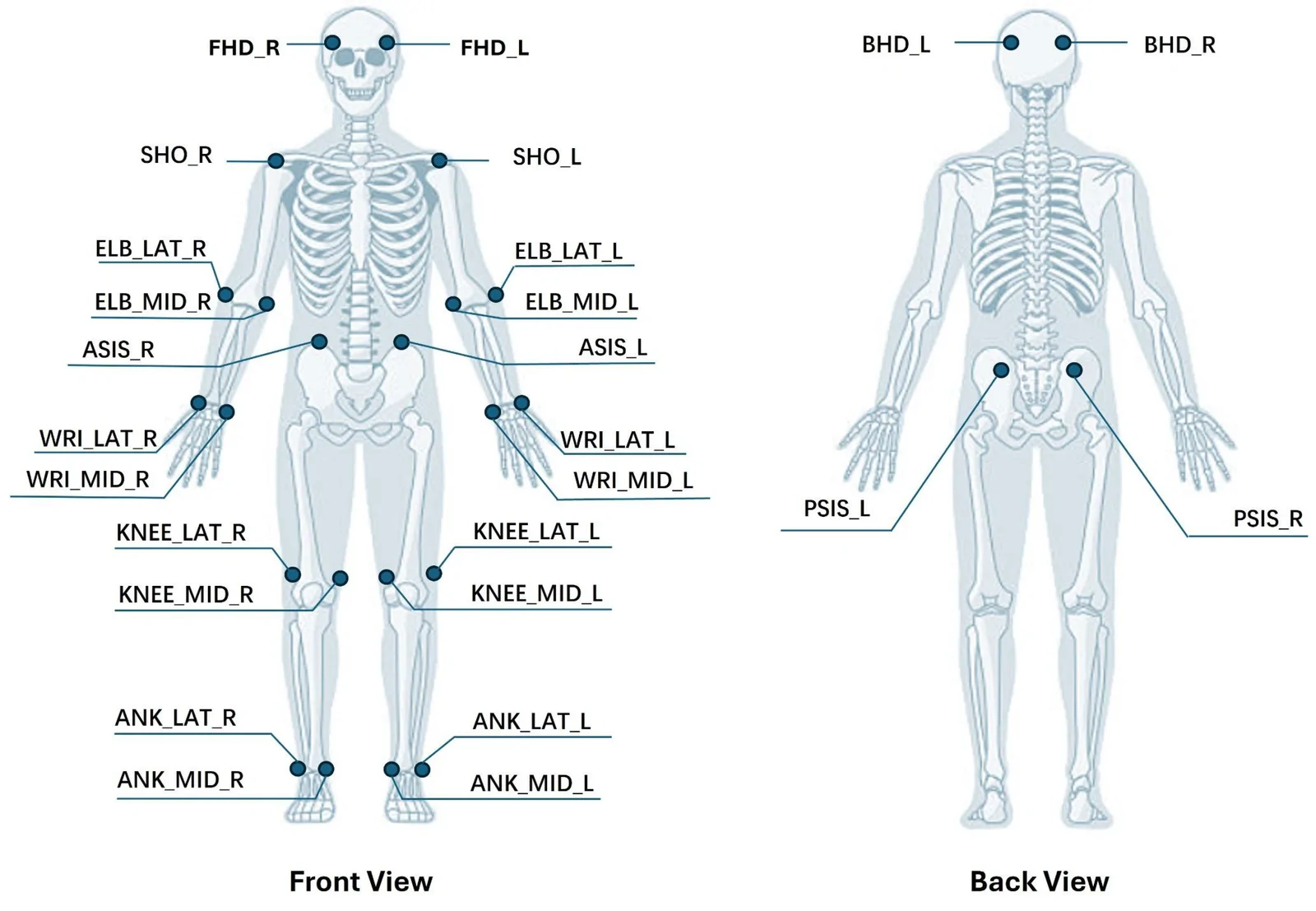
Template of marker arrangement used for the motion capture system, shown in front (**left**) and back (**right**) views. Marker labels use the following abbreviations: FHD (forehead), BHD (back of head), SHO (shoulder), ELB (elbow), WRI (wrist), KNEE (knee), and ANK (ankle). The suffixes L and R denote left and right sides of the body, respectively, while LAT and MID indicate lateral and medial marker positions. This marker configuration is designed to enable consistent alignment between motion capture data and camera-based human pose estimation.

The participant was equipped with a full-body set of 26 retro-reflective markers. To ensure compatibility with the detected joints’ positions in the 2D human pose estimation method, e.g., RTMO [20], we created our own body template with the specified positions of the markers shown in Figure 4. This template covers anatomically meaningful joint locations, including the head, shoulders, elbows, wrists, hip center, knees, and ankles, which are commonly used in human motion analysis and have been shown to be particularly relevant for functional movements, such as turning [4]. 3D marker trajectories were captured at 100 Hz using the Vicon system with its 18 infrared cameras. The data were acquired and processed using Nexus 2.16 software.

**Figure 5:**
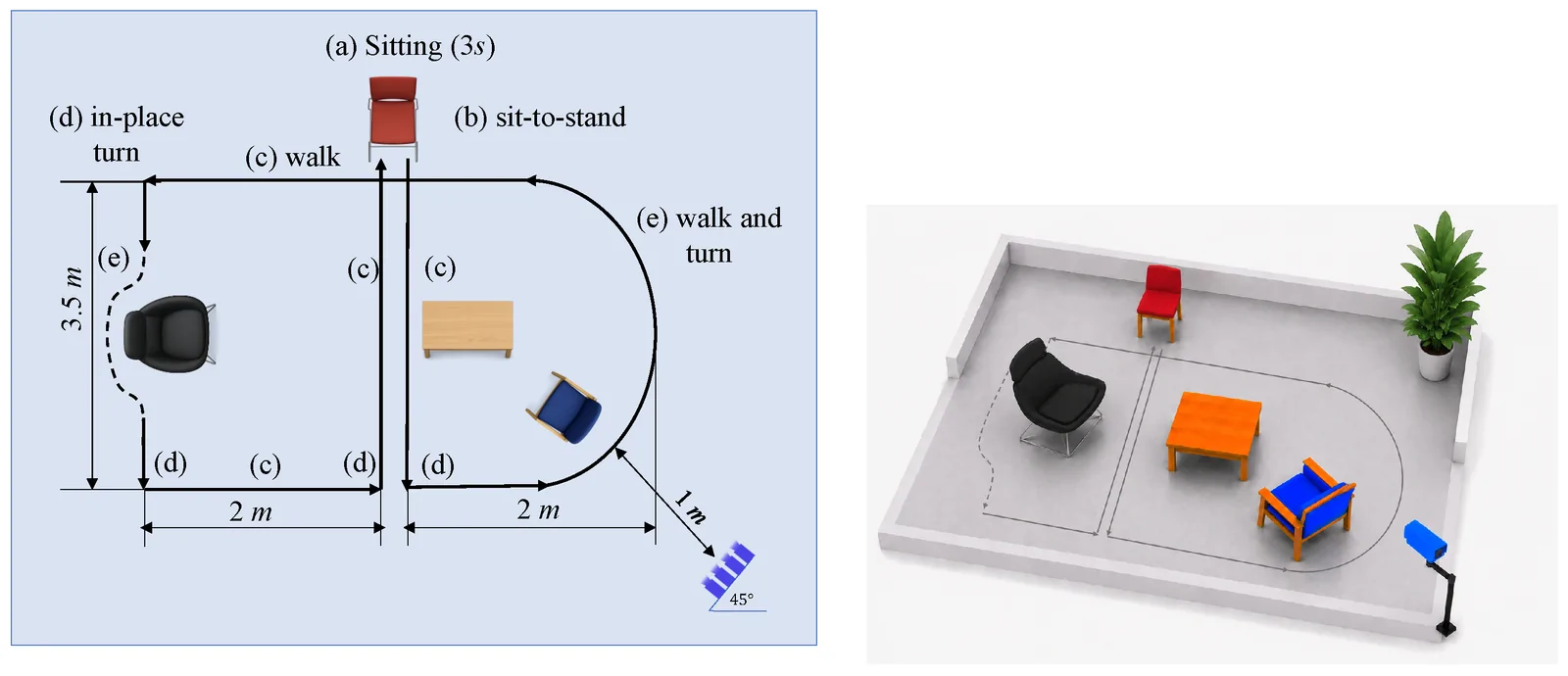
Experimental platform setup and motion task used for evaluating human pose estimation performance. The participant started from the chair and performed a sequence of motion tasks including (a) sitting for 3 s, (b) sit-to-stand, (c) straight walking along the designated path, (d) in-place turning, and (e) walking with a curved turn before returning to the starting point. The layout also shows the spatial arrangement of furniture (e.g., chairs and coffee table), the walking paths, and the camera placement relative to the capture area.

As illustrated in Figure 5, the participant was instructed to follow a predefined path within the experimental area and perform a sequence of functional tasks commonly encountered in daily indoor activities. The task sequence includes sitting, sit-to-stand, walking along straight lines, in-place turning, and continuous walking with directional changes. The participant completed all tasks at a self-selected comfortable pace to reflect natural movement patterns, without external constraints on speed or cadence.

**Figure 6:**
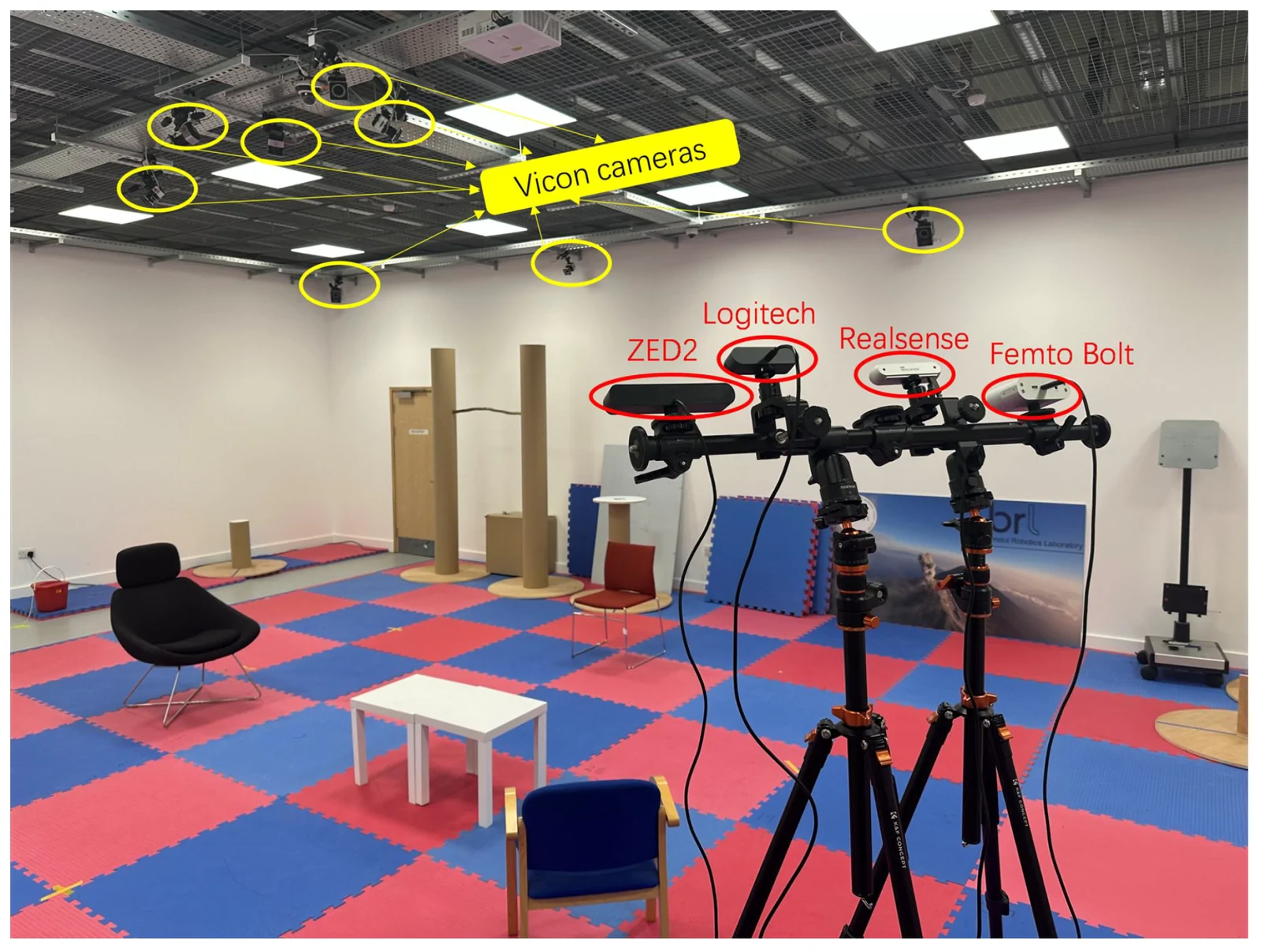
Experimental setup of cameras and Vicon motion capture system.

As shown in Figure 6, the Vicon cameras were distributed around the perimeter of the experimental area to ensure full coverage of the capture volume. The four cameras under investigation were mounted on tripods and oriented toward the capture space.

Three control factors were considered in this experiment: lighting condition, camera location, and occlusion scale, as summarised in Table 2. Two lighting conditions were evaluated, normal lighting (450–600 lux) and low-lighting (50–100 lux). Camera location was examined at three mounting heights with corresponding viewing angles (explained later) selected for each height. Finally, two occlusion conditions were considered: a non-occluded condition without any furniture and an occluded condition with furniture present in the capture area. The participant performed the full motion task once under every combination of these experimental control factors, giving 12 trials per camera (2 lighting conditions × 3 mounting heights × 2 occlusion conditions). All cameras recorded simultaneously, so the same trials were captured by every device.

**Figure 7:**
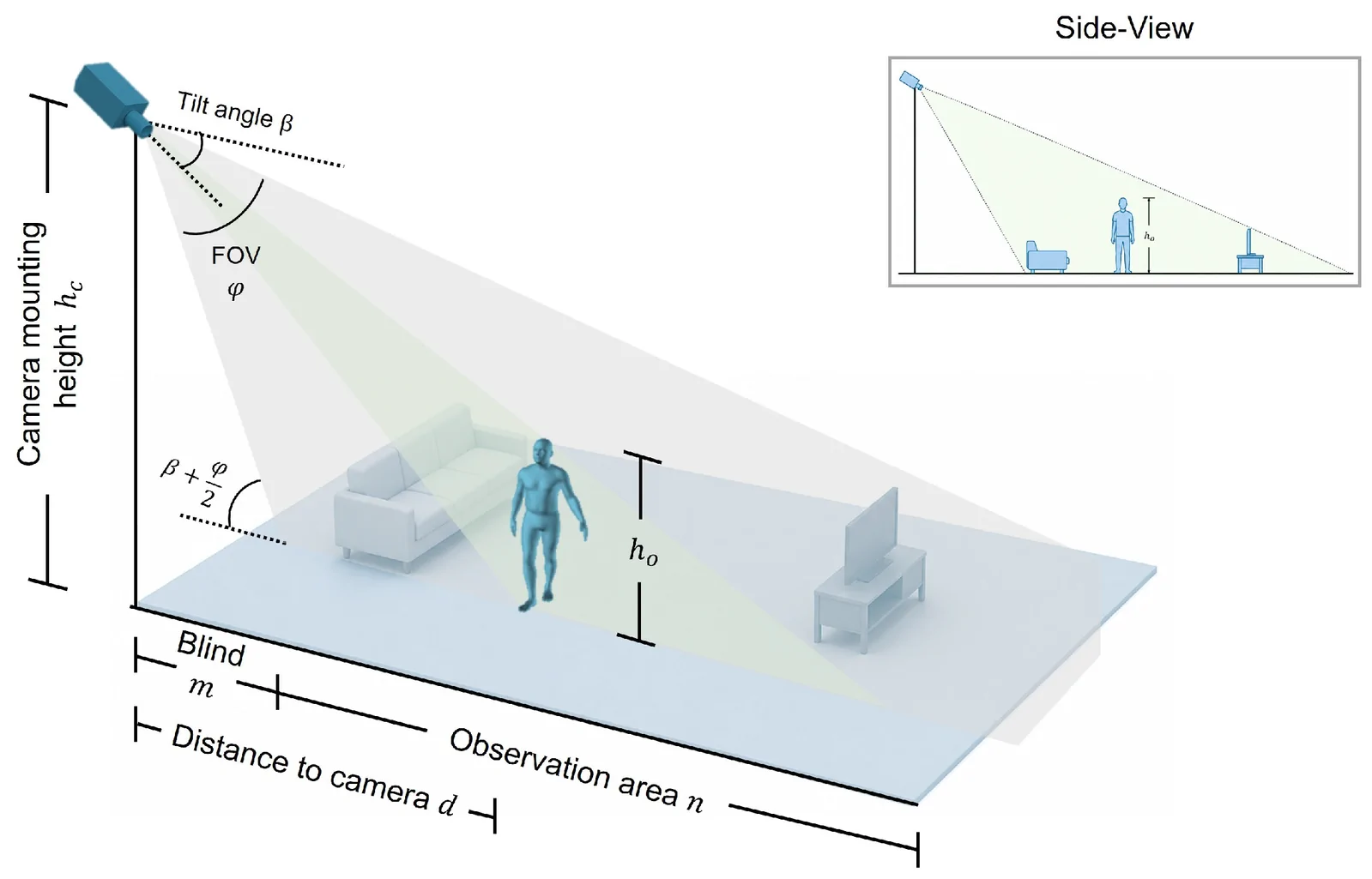
Demonstration of the feasible camera tilt angle range measurement.

To ensure that the full human body remains within the camera field of view, the camera must be positioned at an appropriate tilt angle. For a given camera height, the tilt angle is determined by the requirement that the field of view fully contains both the subject’s head and feet, corresponding to the limiting cases where the head or feet lie exactly on the field-of-view boundaries. Based on this geometric relationship, as illustrated in Figure 7, the allowable tilt angle range is defined by corresponding upper and lower bounds. Specifically, the camera tilt angle β is restricted to

(7) $$\beta_{lower}-\frac{\varphi }{2}<\beta <\beta_{upper}+\frac{\varphi }{2},$$

where $\beta_{lower}=\tan^{-1}\left(\frac{h_{c}}{d}\right)$ and $\beta_{upper}=\tan^{-1}\left(\frac{h_{c}-h_{o}}{d}\right)$. Here, $h_{c}$ denotes the camera mounting height, $h_{o}$ the subject height, *d* the horizontal distance between the camera and the subject, and φ the vertical field of view of the camera. The distance *d* is assumed to satisfy m≤d≤m+n, where *m* and m+n denote the near and far boundaries of the expected observation region, respectively. In the experimental setup, the tilt angle ranges are computed for each camera at three mounting heights (1.8 m, 2.0 m, and 2.2 m), which are selected to reflect typical indoor installation scenarios, such as wall-mounted or elevated camera placements in residential and clinical environments. The subject height is set as $h_{o}$ = 1.8 m, and the camera-to-subject distance is assumed to lie within the interval 1.5 m ≤ *d* ≤ 5.0 m, covering typical indoor interaction distances. For each camera and mounting height, the corresponding feasible tilt angle range is calculated using Equation (7), and the recommended tilt angle is defined as the midpoint of this range. This choice provides a representative tilt angle within the feasible range, providing a balanced margin with respect to both the upper and very low visibility constraints.

The resulting angle ranges and recommended tilt angles are summarised in Table 3. For the Femto Bolt at a mounting height of 2.2 m, there is no solution for the computed range due to field-of-view limitations. To maintain consistency across cameras, a tilt angle of $30^{\circ }$ is adopted for this configuration. Although this angle lies outside the theoretically feasible range, it still keeps most of the subject within the camera field of view.

#### 3.2.3. Signal Processing and Synchronisation

Data post-processing includes labelling marker trajectories automatically to start with and then inspecting them to ensure consistency. Temporal gaps in the marker data caused by occlusions were then manually filled within the Nexus software 2.16 (Vicon Motion Systems Ltd., Oxford, UK) to reconstruct the full-body motion model.

The RGB streams of all cameras operated at a frame rate of 30 fps and were used for the 2D pose evaluation. As the Femto Bolt supports depth acquisition at a maximum of 15 fps in its full-resolution (1024×1024) mode, the depth streams of all three RGBD cameras were recorded at 15 fps so that the 3D evaluation was performed under a common frame rate across devices. The Vicon motion capture system recorded marker trajectories at 100 Hz. Temporal synchronisation between the camera and the Vicon system was achieved by aligning their temporal references using a trigger-based event (a clap action), followed by timestamp alignment and interpolation. The synchronisation was applied separately to the RGB and depth streams using their respective frame rates. Further details of the synchronisation procedure are provided in Appendix C.

#### 3.2.4. Spatial Alignment of Skeleton Data

We calibrated the cameras’ intrinsic parameters and the extrinsic parameters between each camera and the Vicon system, respectively. The estimated extrinsic parameters were used to establish the spatial alignment between the RGB/RGBD cameras and the Vicon coordinate system. See Appendix D for details.

**Figure 8:**
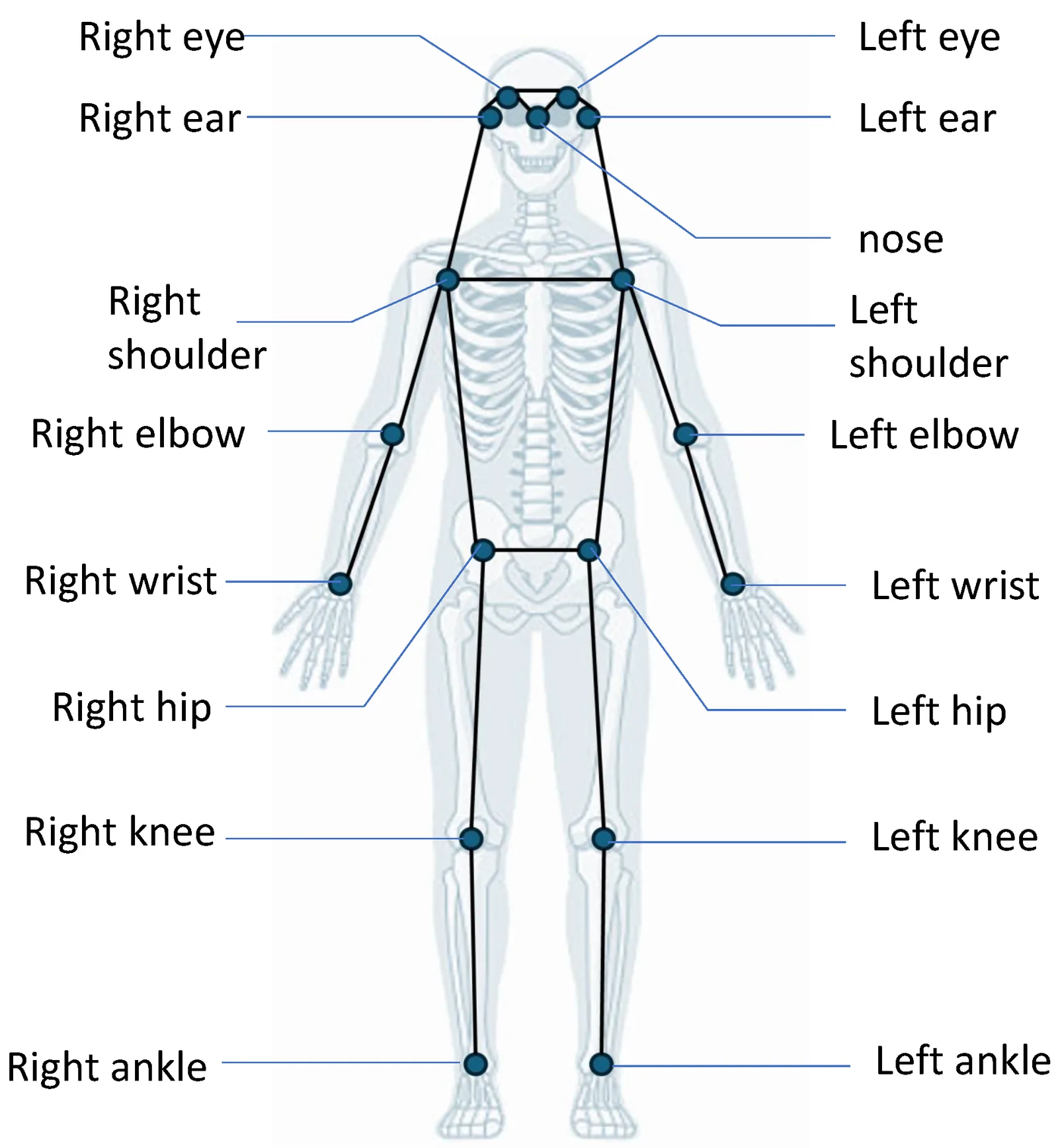
COCO style 2D human pose representation [61].

To enable a consistent comparison between camera-based pose estimation and motion capture data, joints defined in the Vicon marker-based template are mapped to COCO-style skeleton joints [61], as  illustrated in Figure 8 and summarised in Table 4. In particular, the left and right hip joint centers are not directly available in the mocap data. Following the approach described in [62], the hip joint locations are estimated using the posterior and anterior superior iliac spine markers ($PSIS_{R}$, $PSIS_{L}$, $ASIS_{R}$, $ASIS_{L}$) provided by the Vicon system. In Table 4, mid(x,y) denotes the midpoint between joints *x* and *y*. This joint alignment procedure ensures anatomical consistency between the camera-based skeleton and the Vicon reference skeleton.

#### 3.2.5. Human Pose Evaluation

For the camera-based data, 2D human joint positions are extracted independently from the same recordings using recent pose estimation methods. We consider several candidate estimators, including MediaPipe [63] and AlphaPose [64], and selected RTMO [20] and YOLO26 [21], which gave the strongest overall performance among the real-time methods in preliminary testing. These two estimators are state-of-the-art, single-stage, real-time, and optimised for efficient inference on edge hardware (e.g., NVIDIA Jetson platforms), making them well suited for continuous in-home healthcare monitoring. Note that they differ in their underlying design: RTMO is a bottom-up one-stage method, whereas YOLO26 couples person detection and keypoint regression in a single end-to-end network. Both estimators were used with their publicly released pre-trained weights and default architectures and hyperparameters, without any fine-tuning, reflecting realistic off-the-shelf deployment. The inference implementations and pre-trained checkpoints used for both estimators are listed in Appendix E.

The same depth-lifting procedure described below was applied identically to the 2D keypoints produced by both estimators. For RGBD cameras, the estimated 2D keypoints are first transformed from the image coordinate system to the camera coordinate system using the intrinsic calibration parameters. Depth information is then incorporated to recover the corresponding 3D joint positions. To improve robustness against depth noise and local outliers, the depth value associated with each joint is computed as the median depth within a 3×3 pixel neighbourhood centered at the corresponding 2D keypoint location. In contrast, for RGB cameras, only 2D joint positions are estimated, as no depth information is available to recover 3D joint coordinates.

Reference 3D joint data are collected using the motion capture system and treated as the ground-truth measurements. The 3D joint positions are transformed into the camera coordinate system using the extrinsic parameters.

Following existing studies in human pose estimation, multiple quantitative metrics are adopted to evaluate both 2D and 3D pose estimation performance. For 2D pose evaluation, OKS-based mean average precision (mAP) and percentage of correct keypoints (PCK) are used [61,65]. MAP measures the overlap between predicted and ground-truth keypoints, normalised by the person’s scale, and is calculated across multiple OKS thresholds (from 0.5 to 0.95, at steps of 0.05) to reflect overall localisation precision. PCK measures the fraction of predicted joints that fall within a specific error margin. We use PCK@0.2, which represents the percentage of predicted keypoints that fall within a matching threshold of $0.2\times L_{norm}$. $L_{norm}$ is defined as the distance between the left shoulder and right hip, ensuring the metric remains invariant to the subject’s scale within the image. For 3D pose evaluation, mean per-joint position error (MPJPE) provides a direct measure of error by calculating the average Euclidean distance for all joints between the predicted and ground-truth human pose. In this study, MPJPE is computed directly in the calibrated camera coordinate system, without root translation, scale normalisation, or Procrustes alignment. It therefore reflects absolute metric joint-position error, including depth and the subject’s position relative to the camera. In addition, 3D PCK, denoted as PCK@150 mm in this paper, reports the fraction of joints where the error is below a fixed threshold of 150 mm, offering a measure of the model’s robustness against significant depth or localisation outliers [60,66]. All metrics are computed separately for each pose estimator.

## 4. Results

We provide comprehensive evaluation results of the proposed validation protocols across both sensor-level and application-level perspectives. Specifically, we first analyse the metrological performance of the evaluated cameras, including depth measurement bias and precision, temperature and stability during continuous operation, and field-of-view characteristics. These measurements quantify the fundamental sensing properties of the devices under different environmental conditions. We then evaluate the performance on downstream pose estimation tasks using both 2D and 3D evaluation metrics. For 2D pose estimation, mAP and PCK@0.2 are reported, while for 3D pose estimation we report MPJPE and PCK@150 mm.

### 4.1. Metrological Performance Evaluation

**Figure 9:**
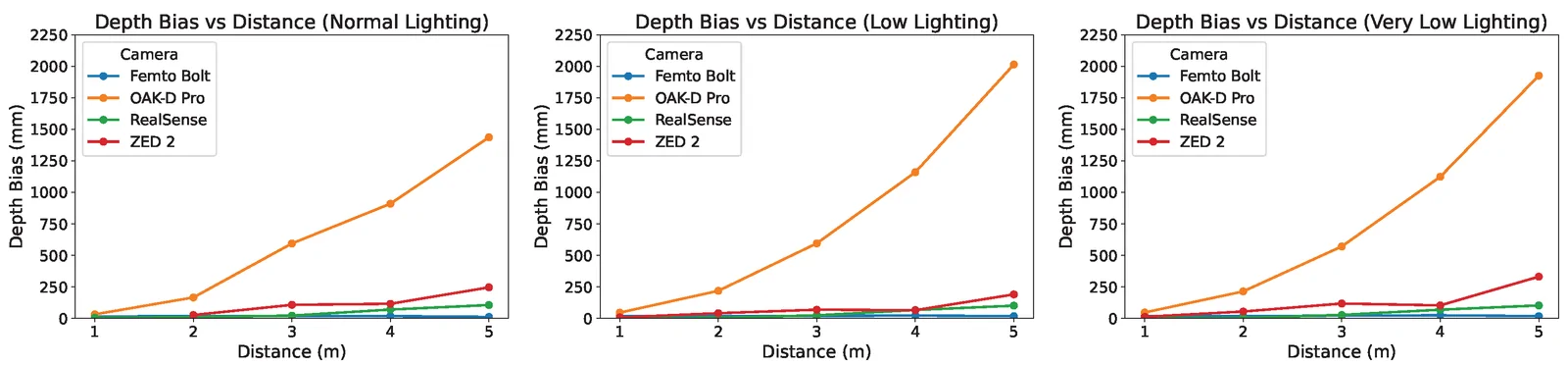
Bias of the tested cameras at different camera-to-target distances under different lighting conditions. The vertical axis represents the depth bias (mm), defined as the absolute difference between the mean measured depth and the ground-truth camera-to-target distance (Equation (1)).

**Depth measurement performance—**Figure 9 illustrates the depth bias as a function of camera-to-target distance under normal-light, low-light, and very low-light lighting conditions for four RGB-D cameras. Across all lighting conditions, while a distance-dependent increase in depth bias is expected, all devices exhibit this trend, except Femto Bolt, which exhibits a markedly different behaviour, with its depth bias remaining nearly constant across the tested distance range, varying only within a narrow range (e.g., within 11.17–17.98 mm under normal lighting, 15.85–21.99 mm under low lighting and 16.52–24.06 mm under very low lighting).

Among the evaluated cameras, OAK-D Pro consistently exhibits the largest bias, with a pronounced monotonic increase as distance grows. This effect is particularly evident beyond 3 m, where the bias rises sharply under all lighting conditions and becomes more severe in low and very low lighting environments. In contrast, Femto Bolt shows the smallest bias across the entire distance range, maintaining relatively stable and low error levels even at longer distances. When averaged over all distances, the bias of Femto Bolt is 15.77 mm under normal-light conditions, 18.27 mm under low-light conditions, and 19.29 mm under very low-light conditions. For RealSense, the depth bias remains very small and comparable to that of Femto Bolt at distances up to 3 m across all lighting conditions. A noticeable increase in bias is only observed beyond 3 m. However, the magnitude of this increase remains relatively limited compared to OAK-D Pro. ZED2 shows a moderate increase in bias with distance that lies between that of the OAK-D Pro and the Intel RealSense, along with a noticeable sensitivity to reduced illumination.

**Figure 10:**
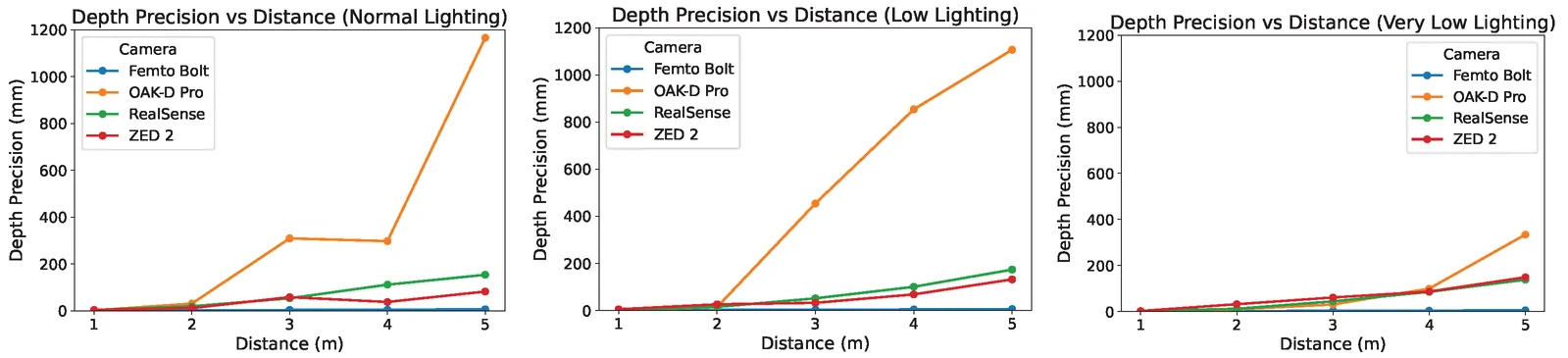
Precision of the tested cameras at different camera-to-target distances under different lighting conditions. The vertical axis represents the depth precision (mm), defined as the standard deviation of repeated depth measurements (Equation (3)).

Figure 10 presents the depth precision as a function of camera-to-target distance under normal, low, and very low lighting conditions for the four evaluated RGB-D cameras. Overall, as expected, depth precision exhibits a clear dependence on measurement distance, with variability increasing as the distance grows, particularly under reduced illumination. However, some interesting observations can be made. Across all lighting conditions, Femto Bolt demonstrates the most stable precision, maintaining consistently low variability over the entire distance range, even at longer distances. In contrast, OAK-D Pro shows a pronounced degradation in precision as distance increases, with a sharp rise beyond 3 m. At a distance of 5 m, the precision of OAK-D Pro is 1165.7 mm under normal-light conditions, 1106.6 mm under low-light conditions, and 334.1 mm under very low-light conditions. The improved precision under very low-light conditions may be attributed to reduced interference from ambient illumination, leading to more stable active depth sensing. RealSense and ZED2 generally exhibit precision values that lie between those of the Femto Bolt and OAK-D Pro. Under very low lighting conditions or at short distances, however, the precision of OAK-D Pro becomes comparable to that of RealSense and ZED2.

As summarised in Table A1, the evaluated cameras employ three different depth sensing principles: ToF, active stereo, and passive stereo. ToF directly estimates depth from the reflected infrared signal and is generally less dependent on scene texture. Correspondingly, the Femto Bolt (ToF) consistently achieves the lowest depth bias and the most stable precision across different distances and lighting conditions. In contrast, both active and passive stereo estimate depth through stereo correspondence, making them more susceptible to matching errors. Passive stereo relies entirely on natural scene texture and illumination, whereas active stereo projects an infrared pattern to improve correspondence in low-texture scenes. Nevertheless, depth accuracy in stereo systems degrades with increasing distance due to reduced disparity resolution [67]. The Intel RealSense D456 and OAK-D Pro both employ active stereo vision, yet their performances differ substantially. Physically, the RealSense D456 features a longer stereo baseline length than the OAK-D Pro, which fundamentally provides higher disparity resolution and smaller depth variance at larger distances [67]. In addition, OAK-D Pro relies on embedded stereo matching on a low-power edge VPU (Luxonis RVC2), while RealSense D456’s depth module is based on Vision Processor D4. The passive stereo-based ZED2 generally exhibits depth measurement performance that lies between those of the Femto Bolt and the OAK-D Pro. As only one ToF and one passive stereo device were tested, these results describe the specific cameras rather than the sensing principles. Notably, the two active stereo devices span the widest performance gap in this study, indicating that device-specific implementation factors can outweigh the sensing principle. Depth performance therefore reflects both the sensing principle and its specific implementation.

**Figure 11:**
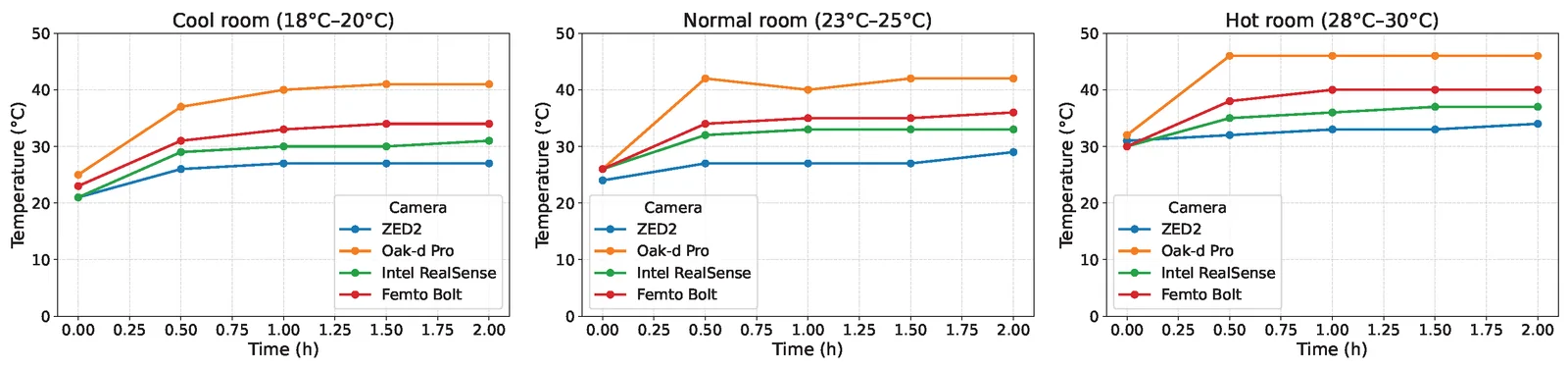
Temperatures of tested cameras after running for 2 h in different room temperatures.

**Temperature and stability performance—**Temperature is evaluated for the four RGB-D cameras under two influencing factors: different operating modes and ambient room temperatures. To examine the impact of ambient temperature, the cameras are operated for two hours under three room temperature conditions: cool (18–20 °C), normal (23–25 °C), and warm (28–30 °C). During this period, the device temperature variations and depth measurement drift are recorded to assess system stability. As shown in Figure 11, across all ambient temperatures, a rapid temperature increase is observed within the first half-hour, followed by a gradual stabilisation phase. OAK-D Pro consistently reaches the highest operating temperature, exhibiting the largest rise across all room conditions. After 2 h of operation, the temperature of the OAK-D Pro reaches 41 °C in a cool room, 42 °C in a normal room, and 46 °C in a hot room. Femto Bolt and RealSense show moderate temperature increases, stabilising at very low absolute temperatures, while ZED2 maintains the lowest and most stable operating temperature. Higher ambient room temperatures lead to elevated steady-state device temperatures for all cameras, although the relative ordering between devices remains consistent across conditions.

**Figure 12:**
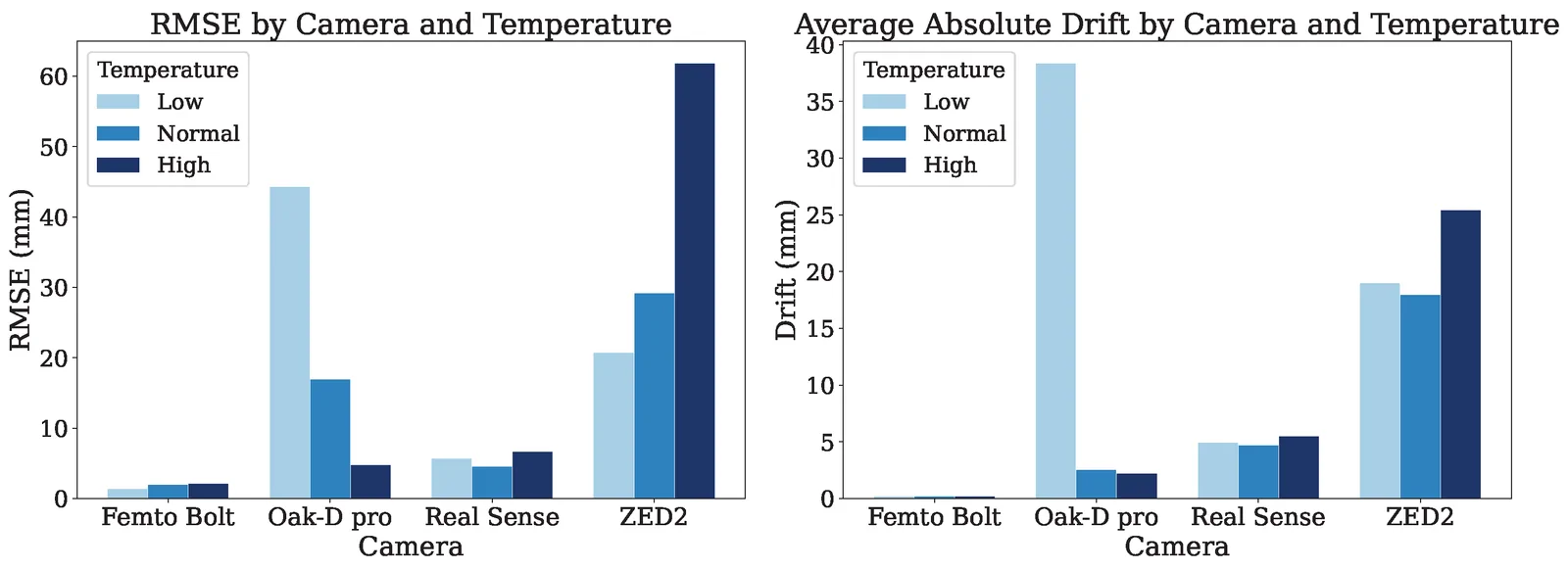
The stability of tested cameras after running for 2 h in different room temperatures.

Figure 12 presents the depth measurement stability of the cameras evaluated at low, normal, and high room temperatures by RMSE and AAD. Femto Bolt consistently exhibits the lowest RMSE and drift across all temperature conditions, indicating high stability with minimal temperature-induced variation. In contrast, OAK-D Pro and ZED2 exhibit substantial increases in both RMSE and drift as temperature rises, suggesting strong sensitivity to temperature variation. RealSense generally shows moderate changes, with RMSE and drift values relatively higher than those of the Femto Bolt.

**Field of view performance—**For FOV assessment, we tested multiple resolution settings to analyse how they affect the measured field of view for each camera. The measured FOV values were compared against the manufacturer’s reported specifications to calculate the relative error, defined as $\left|\mathrm{Measured}-\mathrm{Specified}\right|/\mathrm{Specified}\times 100$, and to verify the accuracy of the manufacturer-supplied parameters. The comparison results are presented in Table 5. The agreement between measured and manufacturer-specified FOV varies across cameras and resolution settings. While Femto Bolt, RealSense and Logitech generally exhibit small relative errors, indicating good agreement with the manufacturer specifications, ZED2 and OAK-D Pro show substantially larger deviations under some resolutions, indicating that manufacturer-specified FOV values are not always consistent with the measured FOV.

**Figure 13:**
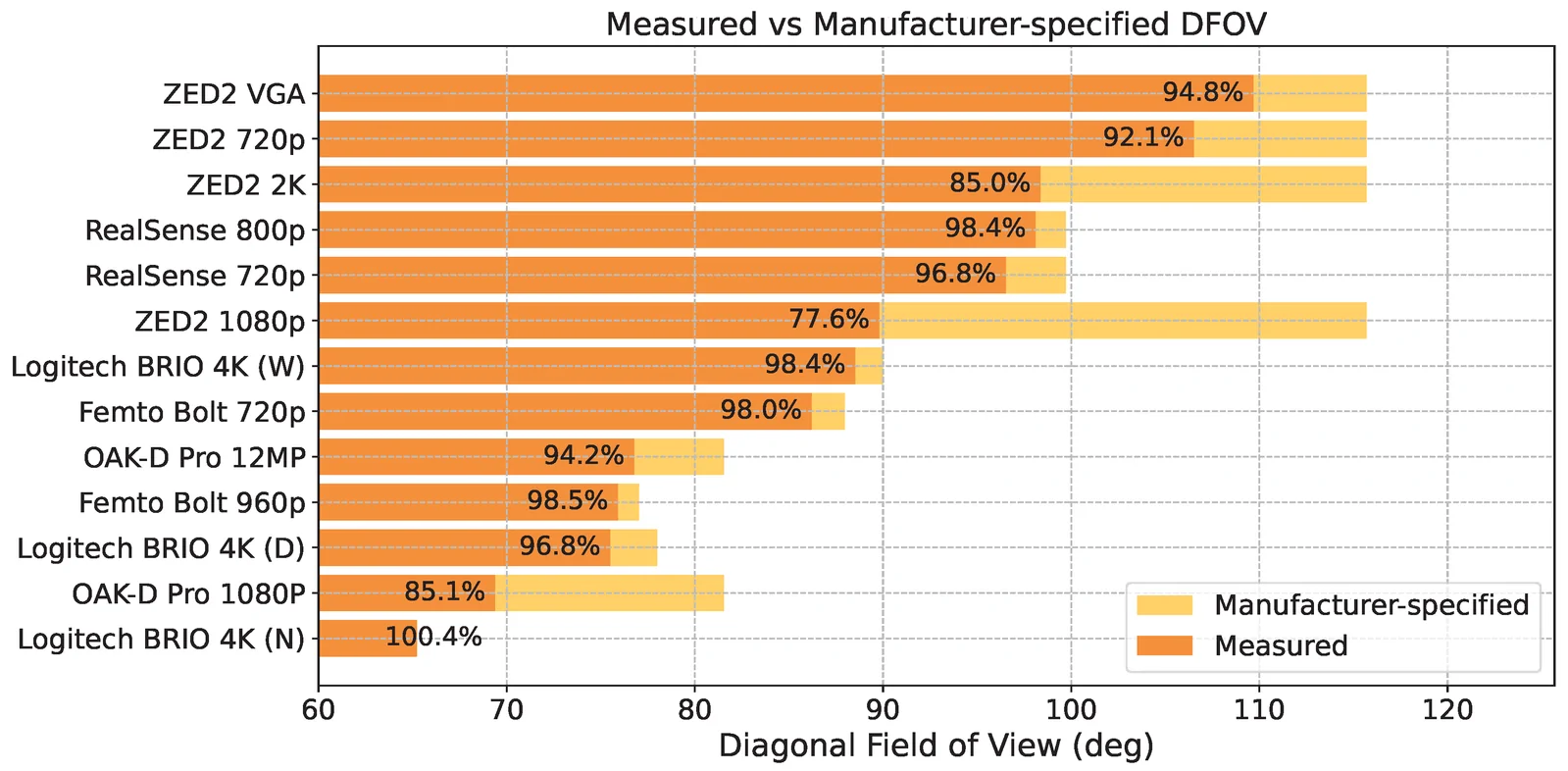
Comparison of measured and manufacturer-specified DFOV across multiple camera models and resolutions. Percentage annotations show how each measurement compares to the corresponding manufacturer reported. Specified DFOV values are derived as described in Section 4.1 (except for the Logitech BRIO 4K, whose manufacturer reports diagonal FOV directly). D: Diagonal, W: Widest, N: Narrowest.

From the FOV evaluations, we observe that most cameras showed slight variations from manufacturer specifications, with the ZED2 showing the largest deviation at some resolutions. The Femto Bolt and RealSense cameras demonstrated the most consistent and accurate FOV measurements relative to their specifications across different resolutions. Although the optical field of view is primarily determined by the lens, the experimentally measured field of view may vary across output resolutions due to different sensor readout strategies (e.g., cropping, ROI selection, or pixel binning). To ensure a fair and aspect-ratio-independent comparison across cameras, we further assess the diagonal field of view (DFOV) for each device. The DFOV is obtained from the horizontal and vertical field-of-view angles defined in Equation (6) as $\tan\left(\theta_{D}/2\right)=\sqrt{\tan^{2}\left(\theta /2\right)+\tan^{2}\left(\varphi /2\right)}$, where $\theta_{D}$ denotes the diagonal field-of-view angle. The same relation is applied to the manufacturer-specified horizontal and vertical values so that measured and specified diagonals are computed on a common basis. The manufacturer of the Logitech BRIO 4K reports the diagonal field of view directly, so its specified value is used as published. The quantitative results are provided in the 3rd column of Table 5, and Figure 13 presents a visual comparison between the measured DFOV and manufacturer specifications. More detailed comparisons of the cameras’ horizontal field of view (HFOV) and vertical field of view (VFOV) with their corresponding manufacturer specifications are presented in Appendix B.

When comparing all cameras at the standard resolution of 1280 × 720, the field of view ranking from widest to narrowest is given in Table 6. ZED2 exhibits the largest measured DFOV, followed by the RealSense and Logitech BRIO 4K, while the Femto Bolt and OAK-D Pro provide comparatively narrower fields of view.

**Figure 14:**
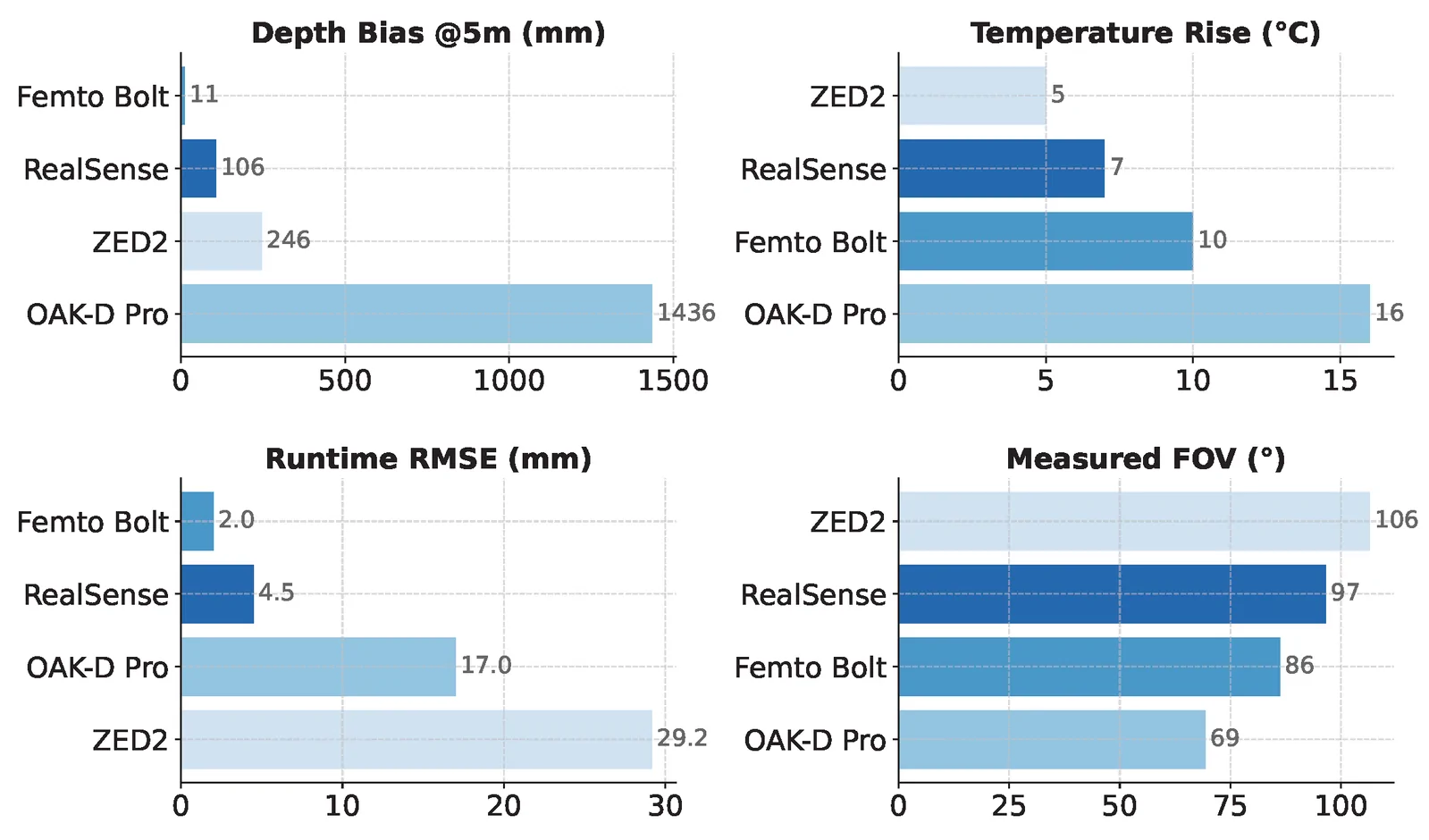
Comparative evaluation of the four depth cameras across key performance metrics: depth bias @ 5 m (normal lighting), temperature rise (normal room), runtime RMSE (normal room) and Measured DFOV at 1280 × 720 resolution. For depth bias, temperature rise, and runtime RMSE, lower values indicate better performance. In contrast, higher values are preferable for measured FOV.

**Figure 15:**
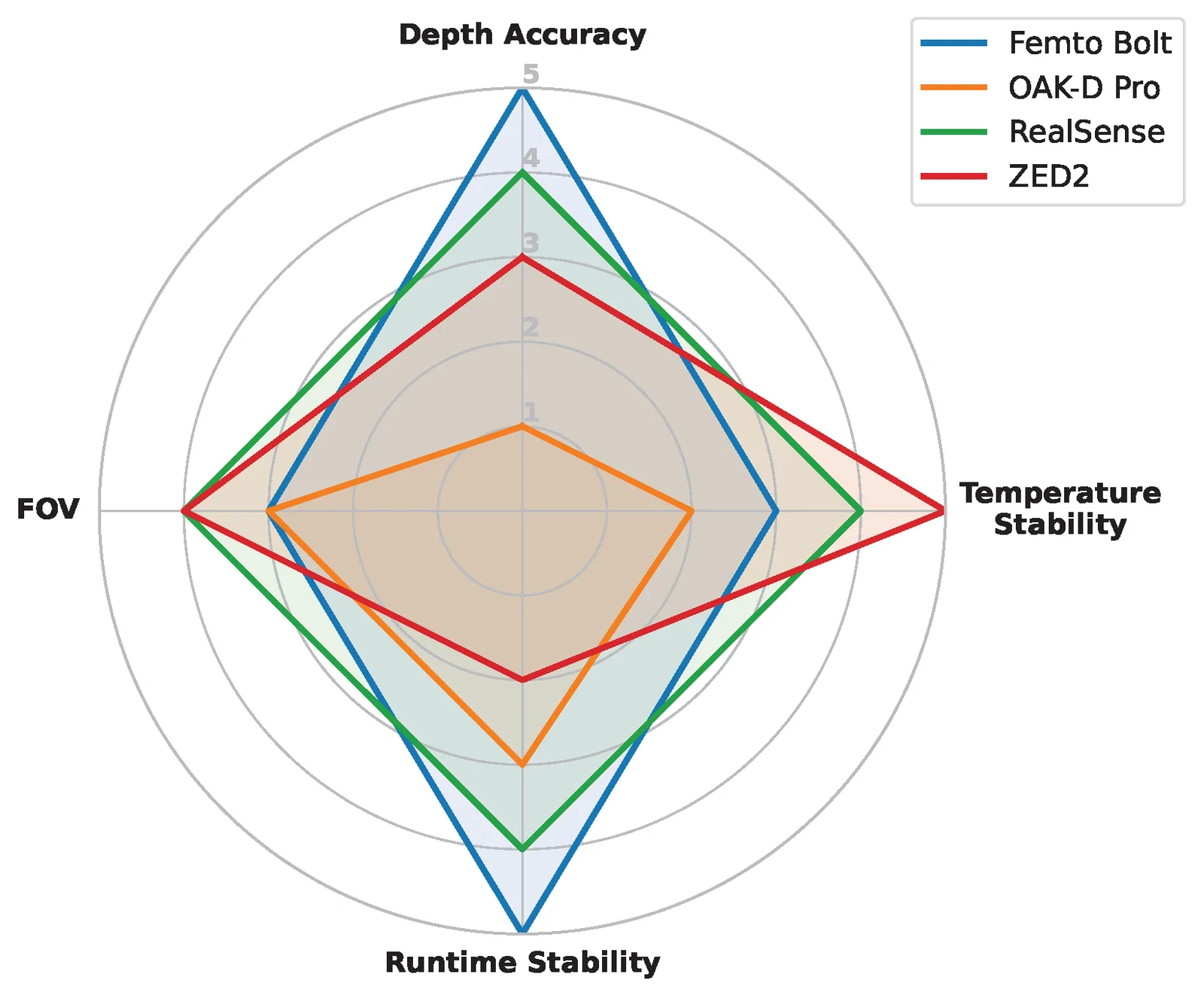
Overview of a qualitative score comparison for the four depth cameras.

**Summary—**To facilitate a clear comparison, we combine the four key metrological evaluations, i.e., depth accuracy, temperature and stability, runtime, and FOV across all four depth cameras. Figure 14 presents a quantitative comparison across these metrics (lower is better for depth bias, temperature rise, and runtime RMSE; a larger FOV is preferred), and Figure 15 provides a qualitative comparison based on relative performance ranks. Overall, Femto Bolt and RealSense achieve the most balanced performance across all metrics, whereas OAK-D Pro and ZED2 show trade-offs between accuracy, stability, and field of view.

### 4.2. Accuracy in Pose Estimation

Following established evaluation practice in human pose estimation [20], OKS-based mean average precision (mAP) and percentage of correct keypoints (PCK) are used to assess 2D pose estimation performance. For 3D pose evaluation, mean per-joint position error (MPJPE) and 3D PCK are employed. All metrics are computed independently for both estimators RTMO and YOLO26 (see Section 3.2.5). As the protocol targets indoor healthcare monitoring where everyday objects may partially obstruct the camera’s view, performance metrics are computed for each combination of camera, lighting, and occlusion conditions, and then averaged using the arithmetic mean across three camera heights (1.8 m, 2.0 m, and 2.2 m), selected to represent practical wall-mounted installation positions in typical residential environments where standard ceiling heights are approximately 2.4 m. As demonstrated by the field-of-view analysis in Section 3.2.2 and Table 3, these heights ensure that a feasible tilt angle exists for most cameras to capture the full body of a standing subject. This aggregation strategy isolates the effects of lighting and occlusion from camera placement, which is examined separately in the height-specific analysis.

**Figure 16:**
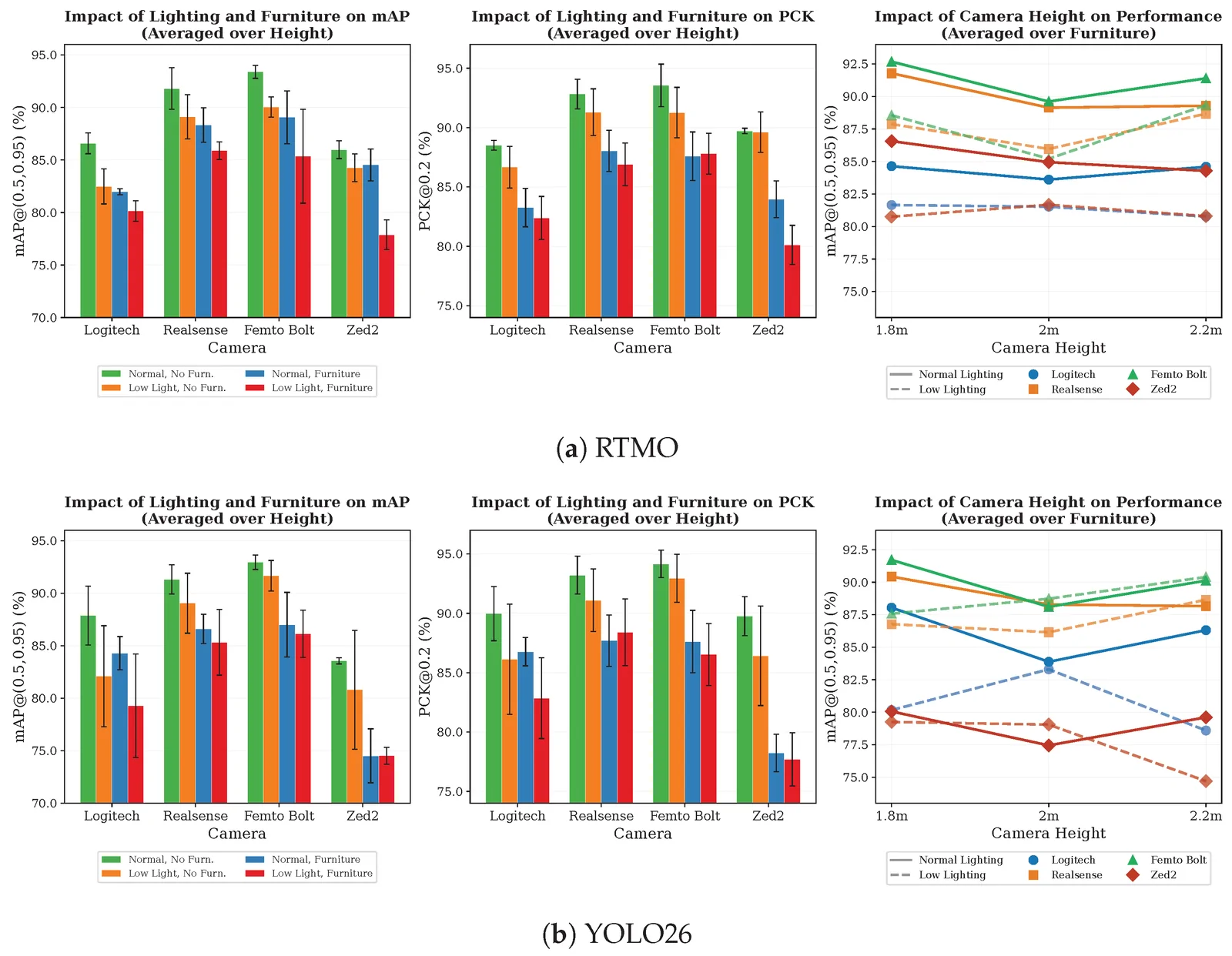
2D pose estimation performance across the four cameras for two pose estimators: (**a**) RTMO and (**b**) YOLO26. In each row, the left and centre panels show the impact of lighting and furniture occlusion on mAP@(0.5,0.95) and PCK@0.2 respectively (averaged over mounting height), and the right panel shows the impact of camera mounting height on mAP (averaged over furniture). Error bars in the left and centre panels show the standard deviation across the three camera mounting heights (n = 3).

**2D pose estimation performance—**Figure 16 presents 2D pose estimation performance obtained by applying RTMO and YOLO26 to the RGB streams captured by each camera, where across all four cameras, the highest mAP and PCK values are consistently observed under normal lighting, and without furniture, as expected. The two estimators produce a broadly consistent ranking across the camera inputs. With RTMO, the highest mean mAP is obtained on the Femto Bolt stream (89.5%), followed closely by the RealSense stream (88.8%). YOLO26 shows a similar trend, with mean mAP values of 89.4% and 88.1% for the Femto Bolt and RealSense streams, respectively. For both estimators, higher accuracy is generally achieved on the Femto Bolt and RealSense streams than on the Logitech and ZED2 streams across the evaluated conditions. Under the most challenging scenario (low lighting with furniture), both estimators show a clear degradation in performance across all camera inputs. With RTMO, mAP values decrease to ~85–86% for the Femto Bolt and RealSense streams, and ~78–80% for the Logitech and ZED2 streams. For YOLO26, the same pattern is observed, with mAP values of ~85–86% for the Femto Bolt and RealSense streams and ~75–79% for the Logitech and ZED2 streams. For the Logitech stream, RTMO achieves performance comparable to the ZED2 stream, with mean mAP values of 82.8% and 83.2%, respectively. For YOLO26 however, the Logitech stream achieves a mean mAP of 83.4% and clearly outperforms the ZED2 stream at a mean mAP of 78.4%, indicating that YOLO26 is more sensitive than RTMO to the visual characteristics of the ZED2 RGB stream, potentially reflecting camera-specific properties such as output resolution, field of view, image sharpness, or noise under the evaluated indoor conditions.

In summary, reduced illumination leads to a measurable decline in 2D pose estimation accuracy across all camera inputs for both estimators. For RTMO, the mAP drop when moving from normal to low lighting ranges from ~2.5 to 4.5%, depending on the camera stream. For YOLO26, the reduction is generally smaller when applied to the three RGBD camera streams, at ~1–2%, suggesting that YOLO26 is less affected by reduced illumination for these inputs. The main exception is the Logitech RGB stream, for which YOLO26 shows a larger decrease of ~5.4%, indicating that the effect of lighting depends on both the estimator architecture and camera-specific RGB output characteristics, such as light sensitivity, resolution, and image quality. The introduction of furniture further reduces performance, with mAP drops of 3.0–4.5% for RTMO and 3.0–7.7% for YOLO26. For YOLO26, the largest occlusion-related reductions are observed on the ZED2 and Femto Bolt streams, suggesting greater sensitivity of this estimator to occlusion for these particular camera outputs.

Camera mounting height has only a minor influence on 2D pose estimation within the evaluated range for both estimators. As shown in the rightmost panel of Figure 16, mAP values remain relatively stable across the three mounting heights for all camera inputs under both lighting conditions, with variations typically below ~3.5%. For YOLO26, the variation remains below ~2.5% for every camera stream. This suggests that, within the tested height range, mounting height is not a dominant factor affecting 2D pose estimation accuracy.

**Figure 17:**
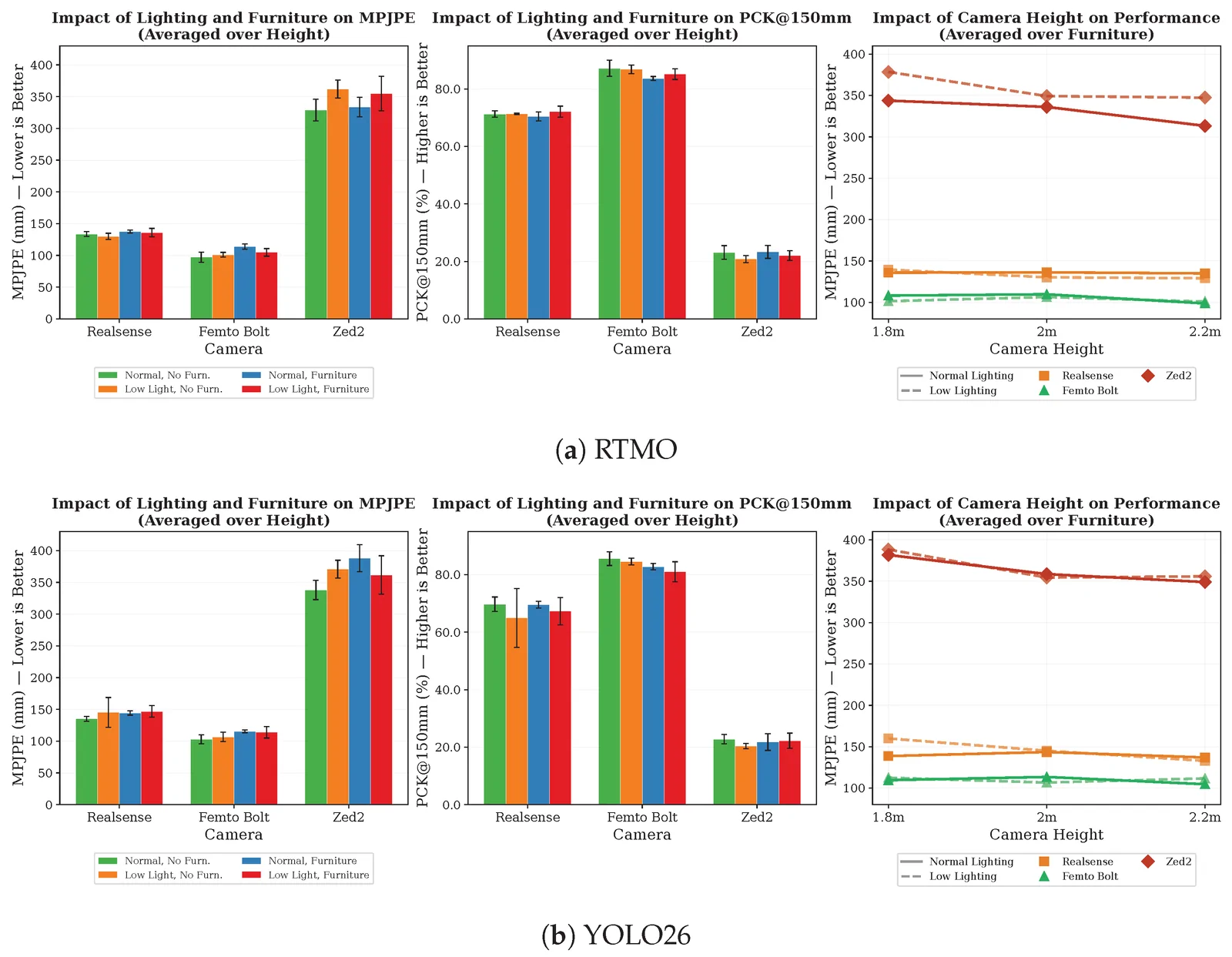
3D pose estimation performance across the three RGBD cameras for two pose estimators: (**a**) RTMO and (**b**) YOLO26. Within each row, MPJPE and PCK@150 mm are reported against lighting and furniture occlusion in the left and centre panels respectively (averaged over mounting height), while the right panel reports MPJPE against camera mounting height (averaged over furniture). Lower MPJPE is better. Error bars denote the standard deviation over the three mounting heights (n = 3).

**3D pose estimation performance—**Figure 17 shows the corresponding 3D pose estimation results for the three RGBD camera streams. The 3D pose accuracy varies substantially across camera inputs, reflecting differences in the underlying depth streams, with the same device ordering and comparable error magnitudes observed for both RTMO and YOLO26. With RTMO, the lowest error is obtained for the Femto Bolt stream, with a mean MPJPE of 104 mm and a mean PCK@150 mm of 85.7%, indicating consistently reliable 3D pose estimation across the evaluated conditions. The RealSense stream shows moderate performance, with a mean MPJPE of 134 mm and a mean PCK@150 mm of 71.3%, while the ZED2 stream has substantially higher error, with a mean MPJPE of 345 mm and a mean PCK@150 mm of 22.3%. YOLO26 yields similar figures and the same ordering: Femto Bolt at a mean MPJPE of 110 mm and mean PCK@150 mm of 83.5%, RealSense at 143 mm and 67.9%, and ZED2 at 365 mm and 21.8%. The close correspondence between the two architecturally distinct estimators supports the interpretation that these differences are mainly associated with the camera-specific depth streams rather than estimator-specific behaviour.

The impact of environmental conditions is more pronounced for 3D than for 2D evaluation, although for Femto Bolt and RealSense the absolute changes remain small relative to their baseline error, and the largest effects are observed on the ZED2 stream. Under normal lighting without furniture, all three cameras generally achieve their best 3D performance. The two estimators agree on the device ordering, but differ in how the ZED2 stream responds to environmental change, so we report the lighting and furniture occlusion effects for each estimator in turn. The transition to low lighting causes a notable increase in MPJPE for the ZED2 stream under RTMO, by ~27 mm on average over height and furniture, while Femto Bolt and RealSense remain largely unaffected, with average MPJPE changes below 3 mm. Under YOLO26, Femto Bolt again remains essentially unaffected (an average change of about 1 mm) and RealSense shows a modest low-light increase of ~6 mm. For the ZED2 stream, however, the low-light effect is strongly furniture-dependent: MPJPE increases by ~33 mm without furniture but decreases by ~26 mm with furniture, so that the two nearly cancel to an average change of only about 3 mm. This near-cancellation reflects the instability of ZED2’s depth-based 3D estimates under reduced illumination rather than genuine robustness to lighting. A slightly higher PCK@150 mm was observed in a few isolated cases under the furniture condition (e.g., RealSense and ZED2 under low-light conditions); however, the differences were marginal. The no-furniture condition does not imply no occlusion, as body self-occlusion is inherently present during human motion. Moreover, this behaviour was observed only in a few isolated cases rather than representing a systematic trend across cameras or experimental conditions.

Furniture occlusion increases MPJPE on the Femto Bolt stream by ~10 mm on average and on the RealSense stream by ~5 mm, with both effects consistent across the two estimators. For the ZED2 stream, the occlusion effect is again estimator-dependent—it adds little beyond the already high baseline error under RTMO, whereas under YOLO26 it increases MPJPE by ~20 mm on average.

Camera height has limited impact on Femto Bolt and RealSense under both 3D estimators, with MPJPE values remaining relatively stable across the 1.8–2.2 m range (for YOLO26, Femto Bolt varies by less than ~3 mm). ZED2 shows gradual improvement at higher mounting positions (from 361 mm at 1.8 m to 330 mm at 2.2 m under RTMO, and from ~385 mm at 1.8 m to ~353 mm at 2.2 m under YOLO26), though the overall pose estimation error remains considerably higher than that of the other two cameras.

**Summary—**The 2D evaluation demonstrates that all four cameras provide reliable 2D pose estimation under typical indoor conditions, with performance differences across devices being relatively small and the camera ranking preserved across both estimators. The 3D evaluation, however, reveals substantial differences driven primarily by depth sensing quality, again with identical device ordering under RTMO and YOLO26. This is expected because the 3D joint coordinates are reconstructed by combining estimated 2D keypoints with the measured depth values. Since the 2D pose estimators achieve comparable accuracy across cameras, the differences in 3D pose accuracy are primarily attributable to the quality of depth sensing. The correspondence between metrological performance (Section 4.1) and 3D pose estimation accuracy is therefore evident under both estimators. These findings underline that for applications requiring 3D pose information, the quality of the depth sensing modality is a critical factor.

## 5. Discussion and Conclusions

In this study, we proposed two technical validation protocols to evaluate RGB and RGBD camera performance under diverse metrological and indoor environmental conditions, and to assess their suitability for human movement monitoring indoors using pose estimation as a primary representation. The protocols systematically evaluate five cameras (four RGBD and one RGB) in the metrological assessment and four cameras (three RGBD and one RGB) in the pose estimation evaluation, across complementary dimensions: metrological characterisation of depth accuracy, thermal stability, temporal drift, and field of view, and then application-level assessment of 2D and 3D human pose estimation under controlled variations in lighting, camera placement, and occlusion.

The results demonstrate that camera performance is strongly influenced by environmental deployment factors. Reduced illumination and furniture occlusion both degrade pose estimation performance, though their relative impact varies across cameras and evaluation dimensions. In the 2D evaluation, lighting and furniture effects are of broadly comparable magnitude under RTMO, while under YOLO26 the lighting effect is smaller for the RGBD streams than for the RGB-only Logitech stream. In the 3D evaluation, the effect of lighting is both camera- and estimator-dependent. ZED2 is sensitive to reduced illumination under RTMO, while under YOLO26 its low-light response varies with furniture in opposing directions, reflecting unstable depth-based 3D estimation rather than robustness. Femto Bolt and RealSense remain largely robust under both estimators. More generally, device rankings are tight in 2D and widely separated in 3D, which follows from the pipeline described in Section 3.2.5: a camera with adequate RGB but poor depth yields accurate 2D keypoints yet large 3D errors.

The effect of furniture occlusion on 3D pose estimation also varies by camera and lighting condition, with no single pattern applying uniformly across all devices. Camera mounting height, within the 1.8–2.2 m range, has a comparatively minor effect on pose estimation, suggesting that height selection can be guided primarily by field-of-view coverage rather than accuracy considerations.

While it may appear that RGBD camera-based 3D pose estimation yields higher MPJPE than recent RGB image-based 3D human pose estimation models [68,69,70], the two are not directly comparable due to their different evaluation objectives, experimental conditions and sensing mechanisms. First, most monocular methods are trained and evaluated on benchmark datasets such as Human3.6M, which are collected in highly controlled environments. When deployed in more complex real-world scenarios, their performance typically degrades due to the domain gap. In contrast, our evaluation is conducted with varying camera configurations, lighting conditions, and occlusions. Moreover, monocular RGB-based methods recover metric scale from learned priors rather than measuring it, so their absolute accuracy may be affected by scale ambiguity, pathological movement and assistive devices, occlusion, and unseen home environments. Validating them on real patients under these conditions remains future work rather than a substitute for direct depth sensing. Furthermore, our work aims to evaluate the practical performance of commercially available RGBD cameras for continuous healthcare monitoring, rather than developing a new state-of-the-art 3D pose estimation algorithm.

Rather than identifying a single universally optimal device, the findings highlight that camera suitability depends on deployment requirements. This study provides practical guidance for selecting camera categories in healthcare monitoring applications. From an application perspective, modality choice follows the target representation. For tasks relying only on RGB information such as 2D pose estimation, all four cameras, including the RGB-only Logitech, provide comparable detection performance, allowing selection to be driven by cost and integration requirements. When absolute 3D geometry is required (step length, inter-joint distances, joint range of motion), RGBD cameras are recommended and the depth quality is the limiting factor. Among the devices tested, the Femto Bolt provided the highest depth accuracy and the RealSense D456 offered the best overall balance, while the other two stereo devices, i.e., ZED2 and OAK-D Pro, showed relatively larger errors. The substantial performance difference between the two active stereo cameras (RealSense D456 and OAK-D Pro) indicates that depth quality cannot be inferred from sensing technology alone. Users are therefore encouraged to verify the performance of their specific devices using Protocol 1.

Cost follows the same split: the RGB-only Logitech BRIO 4K is naturally lower-priced than the RGBD devices, which occupy a relatively comparable price band. As absolute prices vary by vendor and region and change over time, we do not report them. Table 7 summarises the performance of all cameras across both protocols alongside a recommended deployment scenario for each device. Notably, the device ranking established in Protocol 1 by depth quality carries directly into the 3D pose-estimation ranking in Protocol 2, whereas the field-of-view and thermal characteristics constrain spatial coverage and long-term reliability rather than per-frame accuracy.

Several limitations should be acknowledged. Each sensing principle was represented by only one or two devices, so the results reflect the specific cameras and configurations tested rather than ToF, active stereo, or passive stereo sensing in general. For Protocol 1, the FOV measurement was performed once for each camera–resolution configuration, limiting the assessment of measurement repeatability. For Protocol 2, the pose estimation experiments were conducted with a single participant, ensuring consistency, but limiting assessment of inter-subject variability. The generalisability of these results across participants therefore remains limited and requires further validation with a larger and more diverse participant cohort. Two pose estimation methods, RTMO [20] and YOLO26 [21], were used, selected as state-of-the-art single-stage real-time estimators suited to edge deployment; evaluating two architecturally distinct methods allows the camera-level conclusions to be checked for robustness against estimator-specific behaviour. Other estimation paradigms were not included in the full evaluation: several alternatives were assessed in preliminary experiments, including MediaPipe [63] and AlphaPose [64], but were not retained, and heavier offline or top-down estimators fell outside the real-time scope of this study. Consequently, relative camera performance may still differ under other algorithms. The occlusion conditions were limited to a fixed furniture arrangement, whereas real-world environments naturally exhibit greater variability. Future work should include repeated FOV measurements, extend the evaluation to multiple participants, incorporate additional pose estimation methods, and assess performance under more varied occlusion scenarios.

## Figures and Tables

**Table 1:** Comparison of existing metrological evaluation studies.

Camera Type	Paper	Cameras	Evaluated Metrological Indicators	Environmental Conditions
RGBD cameras	Heinemann et al. [10]	Stereo	Depth bias, precision, NaN ratio, edge precision, and angle-dependent precision	Camera-to-object distance and lighting
	Zennaro et al. [11]	Structured light and Time-of-Flight (ToF)	Depth accuracy, standard deviation, resolution, and point-cloud accuracy	Camera-to-object distance and lighting
	Servi et al. [12]	Stereo and LiDAR	Probing size error, probing form dispersion, distortion error, flat-form distortion error, systematic depth error, flat-object reconstruction quality, and 3D-object reconstruction quality	Camera-to-object distance
	Carfagni et al. [43]	Stereo	Sphere diameter error, form probing error, size probing error, sphere spacing error, flatness error, systematic depth error, and 3D reconstruction accuracy	Camera-to-object distance
	Abdelsalam et al. [45]	Stereo	Depth estimation error, root mean square error, depth-error distribution, and maximum measurable depth	Camera-to-object distance
RGB cameras	Wueller [48]	Digital still cameras	Exposure time, luminance level, output-image black level, noise, resolution, colour fidelity, and texture preservation	Lighting
	Peltoketo [49]	Mobile-phone cameras	Noise, resolution, colour, exposure, and camera speed	Lighting
	Linhares et al. [50]	RGB digital camera	Colour	Scene type and illumination

**Table 2:** Controlled experimental variables and their corresponding levels.

Control Factor	Levels
Lighting condition	Normal lighting
	Low lighting
Camera location	1.8 m
	2.0 m
	2.2 m
Occlusion	No
	Yes

**Table 3:** The resulting tilt angle (°) for the four cameras under three mounted heights.

Camera	ZED2		RealSense		FemtoBolt		Logitech BRIO 4K	
	Range	Avg.	Range	Avg.	Range	Avg.	Range	Avg.
1.8 m	[16, 35]	26	[18, 32]	25	25	25	[25, 26]	25
2.0 m	[19, 37]	28	[21, 34]	28	27	27	28	28
2.2 m	[21, 39]	30	[24, 37]	30	-	30	30	30

- denotes that no feasible tilt angle range exists for this configuration due to field-of-view limitations. A tilt angle of 30° was adopted for consistency across cameras, as described in the text.

**Table 4:** Human skeleton joints alignment across templates.

COCO Style Pose [61]	Pose Template in Vicon System
Left shoulder	SHO_L
Right shoulder	SHO_R
Left elbow	mid(ELB_LAT_L,ELB_MID_L)
Right elbow	mid(ELB_LAT_R,ELB_MID_R)
Left wrist	mid(WRI_LAT_L,WRI_MID_L)
Right wrist	mid(WRI_LAT_R,WRI_MID_R)
Left hip	Bell et al. [62] (PSIS_R,PSIS_L,ASIS_R,ASIS_L)
Right hip	Bell et al. [62] (PSIS_R,PSIS_L,ASIS_R,ASIS_L)
Left knee	mid(KNEE_LAT_L,KNEE_MID_L)
Right knee	mid(KNEE_LAT_R,KNEE_MID_R)
Left ankle	mid(ANK_LAT_L,ANK_MID_L)
Right ankle	mid(ANK_LAT_R,ANK_MID_R)

**Table 5:** Comparison between measured and manufacturer-specified FOV across cameras and resolution settings.

Camera	Setting	Measured FOV (°)			Specified FOV (°)		Relative Error (%)	
		HFOV	VFOV	DFOV	HFOV	VFOV	HFOV	VFOV
ZED2	2K: 2208 × 1242	90.55	59.11	98.37	110	70	17.7	15.6
ZED2	1920 × 1080	81.88	52.25	89.80	110	70	25.6	25.4
ZED2	1280 × 720	99.39	64.89	106.51	110	70	9.6	7.3
ZED2	VGA 672 × 376	102.00	69.96	109.67	110	70	7.3	0.0
OAK-D Pro	1080P	62.21	37.45	69.37	69	55	9.8	31.9
OAK-D Pro	12MP: 4056 × 3040	64.88	50.62	76.77	69	55	6.0	8.0
Femto Bolt	1280 × 720	78.10	49.98	86.19	80	51	2.4	2.0
Femto Bolt	1280 × 960	63.94	50.13	75.90	65	51	1.6	1.7
RealSense	1280 × 720	88.65	57.59	96.52	90	65	1.5	11.4
RealSense	1280 × 800	88.65	62.92	98.11	90	65	1.5	3.2
Logitech BRIO 4K	1280 × 720 (D)	–	–	75.49	78 ^D^		3.2 ^D^	
Logitech BRIO 4K	1280 × 720 (N)	–	–	65.23	65 ^D^		0.4 ^D^	
Logitech BRIO 4K	1280 × 720 (W)	–	–	88.52	90 ^D^		1.6 ^D^	

^D^ Manufacturer reports diagonal FOV only. – denotes unavailable HFOV or VFOV value.

**Table 6:** Camera field-of-view comparison at 1280×720 resolution. Cameras are ranked by diagonal field of view (DFOV) from widest to narrowest.

Rank	Camera	HFOV (°)	VFOV (°)	DFOV (°)
1	ZED2	99.39	64.89	106.51
2	RealSense	88.65	57.59	96.52
3	Logitech BRIO 4K ^1^	–	–	88.52
4	Femto Bolt	78.10	49.98	86.19
5	OAK-D Pro	62.21	37.45	69.37

^1^ Entry shows “– × –, 88.52 (D)”, where dashes denote unavailable HFOV and VFOV values. The manufacturer of the Logitech BRIO 4K reports only the diagonal field of view. Therefore, only DFOV is measured in this work.

**Table 7:** Consolidated summary of camera performance across both protocols and recommended deployment scenarios. Protocol 1 (metrological) values are representative figures from Figure 14 (depth bias, temperature rise) and Table 6 (DFOV at 1280×720); Protocol 2 (pose) values are mean scores under RTMO, with YOLO26 preserving the same device ordering. OAK-D Pro was excluded from Protocol 2 owing to its large depth error, and the RGB-only Logitech provides no depth and hence no 3D pose. Arrows indicate the preferred direction for each metric: ↓ denotes that lower values are better, and ↑ denotes that higher values are better.

Camera	Protocol 1: Metrology			Protocol 2: Pose		Recommended Use/Trade-Off
	Depth Bias↓ @5 m (mm)	Temp. Rise↓ (°C)	DFOV↑ (°)	2D mAP↑ (%)	3D MPJPE↓ (mm)	
Femto Bolt	11	10	86.2	89.5	104	**Best 3D reconstruction.** Preferred when absolute 3D geometry is needed (gait, joint kinematics). Trade-offs: depth recording at 15 fps at 1024×1024.
RealSense D456	106	7	96.5	88.8	134	**Balanced default for 3D.** Low depth bias, wide FOV, depth recording at 30 fps, and broad software support.
ZED2	246	5	106.5	83.2	345	**Wide coverage.** Widest FOV and lowest running temperature, but high depth bias and drift give weak, light-sensitive 3D.
OAK-D Pro	1436	16	69.4	—	—	**Not recommended.** Largest depth error, narrowest FOV and highest running temperature; excluded from Protocol 2.
Logitech BRIO 4K	—	—	88.5	82.8	—	**Suitable for 2D pose estimation.** RGB only; comparable accuracy to RGBD cameras at the lowest cost.

— denotes that the metric is not applicable for that device: OAK-D Pro was excluded from Protocol 2, and the Logitech BRIO 4K is an RGB-only camera and therefore provides no depth-based measurements.

**Figure A1:**
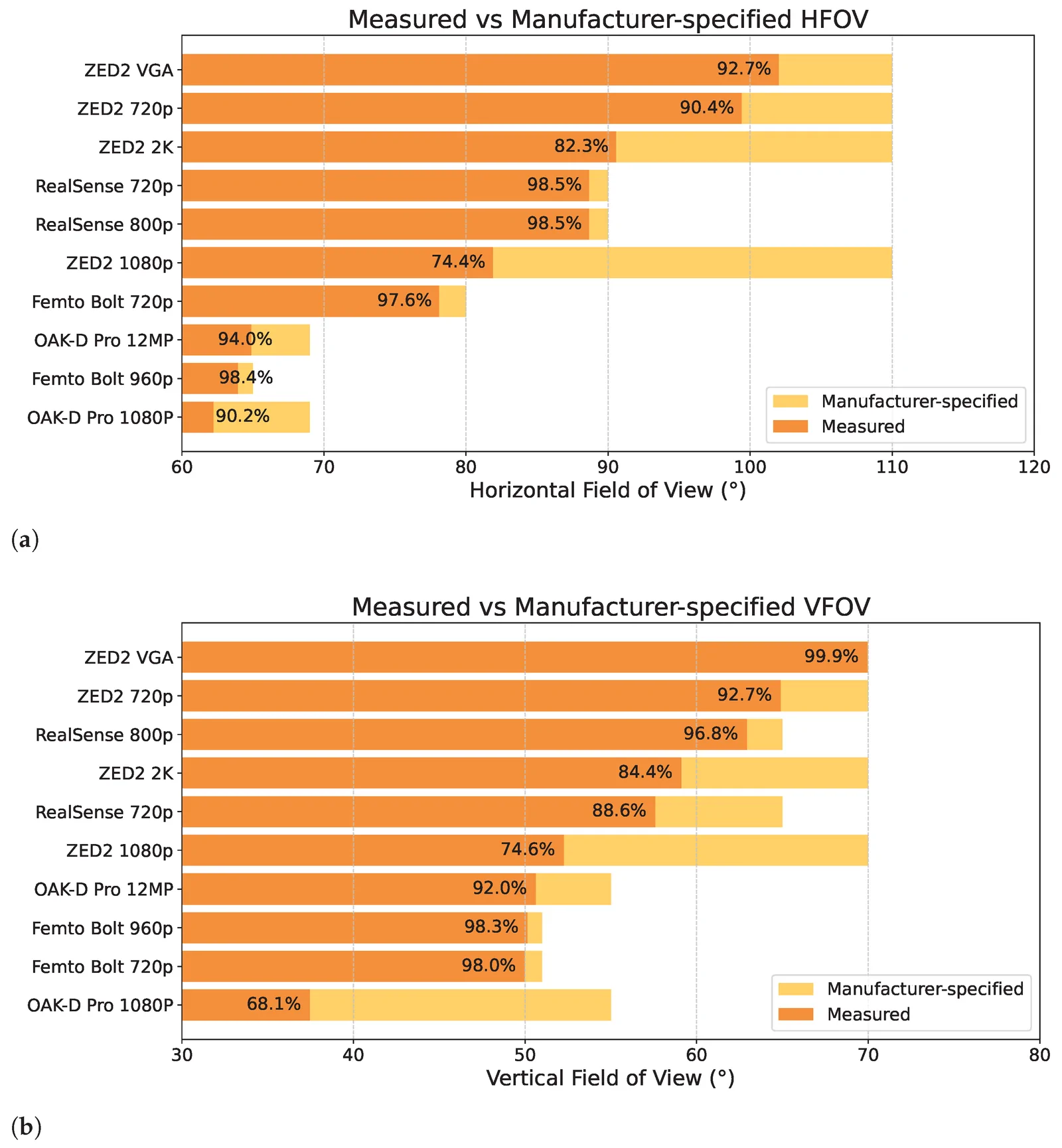
Comparison of measured and manufacturer-specified (**a**) horizontal field of view (HFOV) and (**b**) vertical field of view (VFOV) across multiple camera models and resolutions.

**Figure A2:**
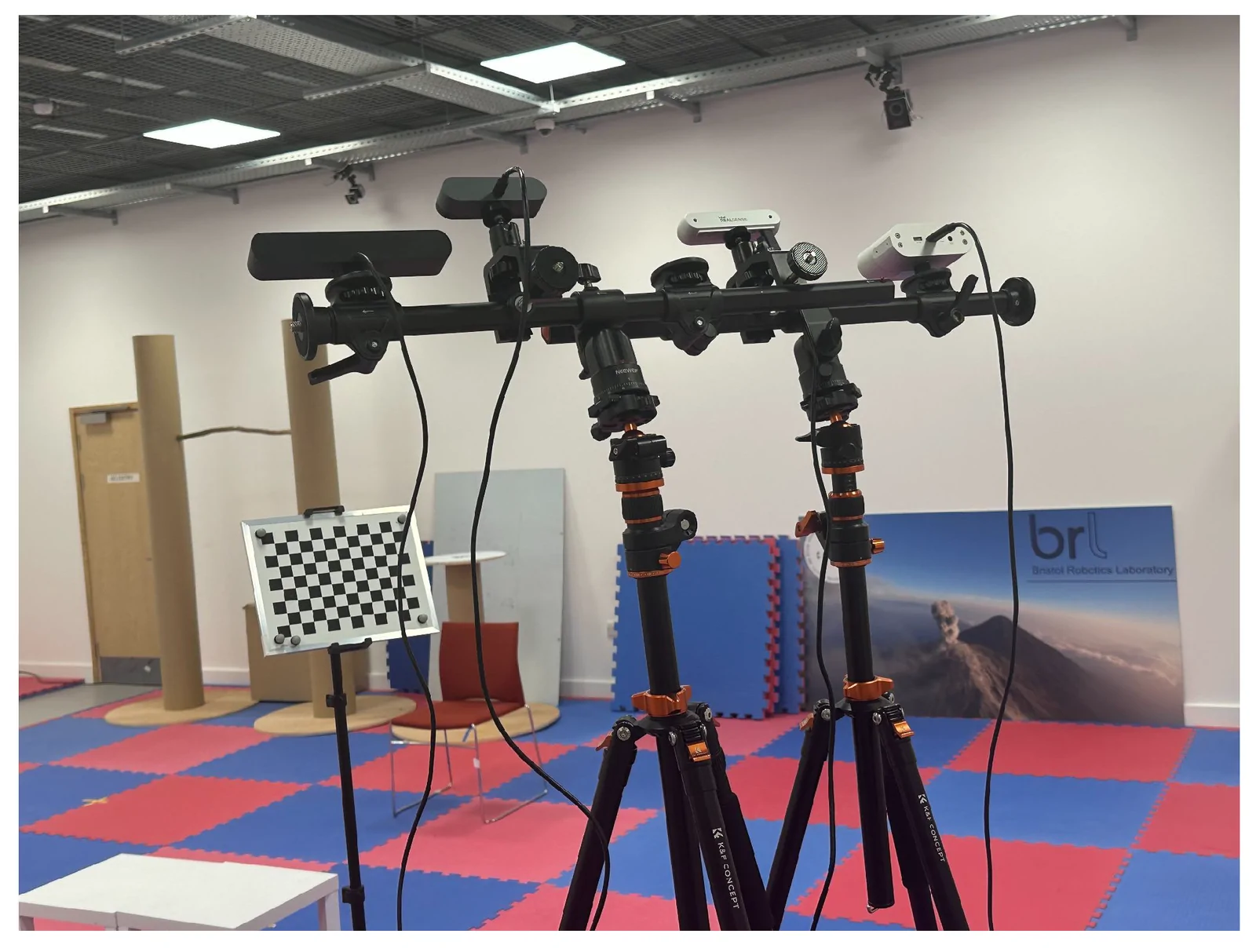
Calibration setup between MoCap and RGB/RGBD cameras.

## Data Availability

The datasets presented in this article are not publicly available due to time limitations. Access to the datasets may be considered upon reasonable request and subject to approval by the authors and the host institution. Requests should be directed to the corresponding author.

## Abbreviations

    The following abbreviations are used in this manuscript:

RGB	Red Green Blue
RGBD	Red Green Blue and Depth
FOV	Field of View
HFOV	Horizontal Field of View
VFOV	Vertical Field of View
DFOV	Diagonal Field of View
ROI	Region of Interest
RMSE	Root Mean Square Error
AAD	Average Absolute Drift
IMU	Inertial Measurement Unit
OKS	Object Keypoint Similarity
mAP	Mean Average Precision
PCK	Percentage of Correct Keypoints
MPJPE	Mean Per-Joint Position Error
MoCap	Motion Capture
ICC	Intraclass Correlation Coefficient
CMC	Coefficient of Multiple Correlation
TUG	Timed Up and Go
FPS	Frames Per Second
ToF	Time-of-Flight

## Appendix A. RGBD Cameras’ Specifications

The specifications of the four evaluated RGBD cameras, including their depth sensing technology, supported resolutions, frame rates, and operating range, are summarised in Table A1. The RGB camera evaluated in this study is the Logitech BRIO 4K (Logitech International S.A., Lausanne, Switzerland).

**Table A1:** Specifications of the evaluated RGBD cameras.

Manufacturer	Name	Depth Technology	Depth Resolution	Depth fps	RGB Resolution	RGB fps	Ideal Range
Orbbec (Troy, MI, USA)	Femto bolt	Time-of-Flight (ToF)	1024×1024	15	3840×2160	30	0.25–5.46 m
			640×576	30	2560×1440	30	
					1920×1080	30	
					1280×720	30	
					1280×960	30	
Intel (Santa Clara, CA, USA)	Realsense D456	Active Stereo	1280×720	30	1280×800	30	0.6–6.0 m
			848×480	90	848×480	60	
			640×480	90	640×480	60	
			640×360	90	640×360	90	
			480×270	90	480×270	90	
			424×240	90	424×240	90	
Stereolabs (San Francisco, CA, USA)	ZED2	Passive Stereo	2208×1242	15	2208×1242	15	0.3–20 m
			1920×1080	30	1920×1080	30	
			1280×720	60	1280×720	60	
			672×376	100	672×376	100	
Luxonis (Littleton, CO, USA)	OAK-D Pro	Active Stereo	1280×800	120	4032×3040	30	0.8–12 m
					3840×2160	30	
					1920×1080	60	
					1280×720	60	

## Appendix B. HFOV and VFOV

The results of HFOV and VFOV of all camera–resolution combinations against their manufacturer specifications are presented in Figure A1.

## Appendix C. Synchronisation

Temporal synchronisation between the RGB/RGBD cameras and the Vicon motion capture system is achieved using a trigger-based event, specifically a clap action performed by the participant within the scene prior to the motion tasks. The clap event is detected by identifying the timestamp corresponding to the minimum distance between the left and right wrists. This timestamp is denoted as $t_{V,\mathrm{clap}}$ for the Vicon system and $t_{C,\mathrm{clap}}$ for the camera. Let $f_{V}$ and $f_{C}$ denote the frame rates of the Vicon system and the camera stream, respectively. As the RGB and depth streams were acquired at different frame rates, the synchronisation was applied separately to each stream, with $f_{C}=30$ Hz for the RGB streams and $f_{C}=15$ Hz for the depth streams. The Vicon timestamps aligned to the camera time reference are then computed as

(A1) $$t_{V\to C}\left(i\right)=t_{C,\mathrm{clap}}+\frac{f_{C}}{f_{V}}\left(t_{V}\left(i\right)-t_{V,\mathrm{clap}}\right).$$

Given the aligned timestamps $t_{V\to C}\left(i\right)$, one-dimensional interpolation is applied to resample the Vicon data, yielding temporally synchronised Vicon trajectories corresponding to the camera frames.

## Appendix D. Intrinsic and Extrinsic Calibration

Intrinsic calibration was performed for all cameras using a standard chessboard-based calibration procedure [71]. For each camera, the extrinsic calibration between the camera coordinate system and the Vicon motion capture coordinate system was then conducted, allowing kinematic data from both systems to be expressed in a common global coordinate frame.

The extrinsic calibration setup employed a chessboard with four retro-reflective markers attached to its outer corners. The chessboard was positioned such that it was simultaneously visible to both the camera and the Vicon system, and synchronised recordings of camera images and marker positions were acquired (see Figure A2). This procedure was repeated ten times with the chessboard placed at different positions and orientations to ensure sufficient calibration diversity. For each time, we recorded the image of the chessboard using the camera and the 3D positions of markers on the chessboard using the Vicon system.

For each recorded image, the 2D locations of the chessboard inner corners were detected using OpenCV’s findChessboardCorners() function. The corresponding 3D coordinates of these corners were inferred from the four outer corner markers captured by the Vicon system, using bilinear interpolation based on the known chessboard geometry. This resulted in a set of 2D–3D point correspondences between the camera image plane and the Vicon coordinate system. The camera extrinsic parameters were subsequently estimated using OpenCV’s solvePnP() function. The estimated rotation matrix R and translation vector t obtained from solvePnP() define the rigid transformation between the Vicon coordinate system and the camera coordinate system. Consequently, each Vicon 3D joint position $P_{v}$ is transformed into the camera coordinate system as

(A2) $$P_{c}=R\cdot P_{v}+t,$$

enabling the transformation of Vicon-based skeleton data into the camera’s coordinate system.

## Appendix E. Pose Estimator Implementations and Checkpoints

Table A2 lists the inference implementations and pre-trained checkpoints used for the two pose estimators. Both were used as publicly released, without fine-tuning. RTMO was run through the rtmlib inference wrapper using the publicly released rtmo-l_16xb16-600e_body7-640x640 checkpoint, and the YOLO26 pose weights (yolo26l-pose.pt) were obtained from the Ultralytics package, while [21] is cited as the architectural reference for YOLO26. Since both checkpoints are publicly distributed and were used without modification, the checkpoint identifiers fully specify the models evaluated in this study.

**Table A2:** Pose estimator implementations and pre-trained checkpoints.

	RTMO	YOLO26
Library	rtmlib	ultralytics
Source	https://github.com/Tau-J/rtmlib (accessed on 24 August 2026)	https://github.com/ultralytics/ultralytics (accessed on 24 August 2026)
Variant	RTMO-l (performance mode)	Large
Checkpoint	rtmo-l_16xb16-600e_body7-640x640	yolo26l-pose.pt
Input size	640×640	640×640
Training data	body7 (7 datasets)	COCO keypoints
